# Chronic kidney disease induces left ventricular overexpression of the pro-hypertrophic microRNA-212

**DOI:** 10.1038/s41598-018-37690-5

**Published:** 2019-02-04

**Authors:** Márta Sárközy, Renáta Gáspár, Ágnes Zvara, Andrea Siska, Bence Kővári, Gergő Szűcs, Fanni Márványkövi, Mónika G. Kovács, Petra Diószegi, László Bodai, Nóra Zsindely, Márton Pipicz, Kamilla Gömöri, Krisztina Kiss, Péter Bencsik, Gábor Cserni, László G. Puskás, Imre Földesi, Thomas Thum, Sándor Bátkai, Tamás Csont

**Affiliations:** 1 Metabolic Diseases and Cell Signaling Group, Department of Biochemistry, Interdisciplinary Excellence Centre, University of Szeged, Dóm tér 9, Szeged, H-6720 Hungary; 2 Laboratory for Functional Genomics, Institute of Genetics, Biological Research Center of the Hungarian Academy of Sciences, Temesvári krt. 62, H-6701 Szeged, Hungary; 3 Department of Laboratory Medicine, Faculty of Medicine, University of Szeged, Semmelweis utca 6, Szeged, H-6725 Hungary; 4 Department of Pathology, University of Szeged, Állomás utca 1, Szeged, H-6725 Hungary; 5 Department of Biochemistry and Molecular Biology, Faculty of Science and Informatics, University of Szeged, Közép fasor 52, Szeged, H-6726 Hungary; 6 Cardiovascular Research Group, Department of Biochemistry, Faculty of Medicine, University of Szeged, Dóm tér 9, Szeged, H-6720 Hungary; 7 IMTTS, Hannover Medical School, Carl-Neuberg Strasse 1, Hannover, 30625 Germany

## Abstract

Chronic kidney disease (CKD) is a public health problem that increases the risk of cardiovascular morbidity and mortality. Heart failure with preserved ejection fraction (HFpEF) characterized by left ventricular hypertrophy (LVH) and diastolic dysfunction is a common cardiovascular complication of CKD. MicroRNA-212 (miR-212) has been demonstrated previously to be a crucial regulator of pathologic LVH in pressure-overload-induced heart failure via regulating the forkhead box O3 (FOXO3)/calcineurin/nuclear factor of activated T-cells (NFAT) pathway. Here we aimed to investigate whether miR-212 and its hypertrophy-associated targets including FOXO3, extracellular signal-regulated kinase 2 (ERK2), and AMP-activated protein kinase (AMPK) play a role in the development of HFpEF in CKD. CKD was induced by 5/6 nephrectomy in male Wistar rats. Echocardiography and histology revealed LVH, fibrosis, preserved systolic function, and diastolic dysfunction in the CKD group as compared to sham-operated animals eight and/or nine weeks later. Left ventricular miR-212 was significantly overexpressed in CKD. However, expressions of FOXO3, AMPK, and ERK2 failed to change significantly at the mRNA or protein level. The protein kinase B (AKT)/FOXO3 and AKT/mammalian target of rapamycin (mTOR) pathways are also proposed regulators of LVH induced by pressure-overload. Interestingly, phospho-AKT/total-AKT ratio was increased in CKD without significantly affecting phosphorylation of FOXO3 or mTOR. In summary, cardiac overexpression of miR-212 in CKD failed to affect its previously implicated hypertrophy-associated downstream targets. Thus, the molecular mechanism of the development of LVH in CKD seems to be independent of the FOXO3, ERK1/2, AMPK, and AKT/mTOR-mediated pathways indicating unique features in this form of LVH.

## Introduction

Chronic kidney disease (CKD) is a clinical syndrome defined as persistent deterioration of kidney function or alteration in kidney structure or both affecting the health of the individual^1–3^. The prevalence of CKD varies between 7–12% in the world^1–3^. The presence of CKD is an independent risk factor for cardiovascular complications^3,4^. Indeed, cardiovascular diseases are the leading cause of morbidity and mortality in all stages of CKD^3,4^. Cardiovascular events are more commonly fatal in patients with CKD than in individuals without CKD^5^. Cardiovascular disease in CKD often presents as HFpEF characterized by left ventricular hypertrophy (LVH) and diastolic dysfunction^1,6^. Later, LVH could contribute to the development of heart failure with reduced ejection fraction, arrhythmias, ischemic heart disease, and sudden cardiac death in CKD^1,6^. LVH is present in 50–70% of CKD patients and up to 90% in dialyzed patients with end-stage renal disease^1,7–10^. Although traditional risk factors, such as hypertension and diabetes mellitus, contribute to high rates of LVH in CKD, the regression of LVH after kidney transplantation suggests other CKD-specific risk factors that remain poorly characterized yet^1,11,12^. Both pre-clinical and clinical studies proved that factors related to CKD itself provoke the development of LVH, regardless of pressure- and volume-overload^13–17^. Therefore, the discovery of specific, so far unexplored mechanisms in the development of LVH is needed to identify novel therapeutic targets for reducing the burden of cardiovascular disease in CKD.

Endogenous microRNAs (miR) are short (approximately 22 bp), non-coding RNA species that are post-transcriptional regulators targeting specific mRNAs, resulting in the suppression of protein synthesis or the increase of mRNA degradation via complementary binding, thus influencing cellular function^18^. miRs have been described as “master switches” in cardiovascular biology^19–22^. The dysregulation of specific miRs has been implicated as key pathological factors in many cardiovascular diseases^19–22^. The miR-212/132 cluster was identified as a central regulator of the development of pressure-overload-induced LVH and heart failure via the repression of the anti-hypertrophic transcription factor FOXO3^23^. Moreover, the overexpression of miR-212 separately from miR-132 was reported to play a role in the development of LVH and heart failure via fetal gene reprogramming in human hearts^24^. Furthermore, the pro-hypertrophic potential of miR-212 was also confirmed in primary neonatal rat cardiomyocytes^25^. Beyond FOXO3, other LVH-associated predicted or validated targets of miR-212 were also identified. These include for instance the extracellular signal-regulated kinase 2 (ERK2)^26^, myocyte enhancer factor 2a (MEF2A)^27^; AMP-activated protein kinase, (AMPK)^28^; heat shock protein 40 (HSP40)^29^; sirtuin 1, (SIRT1)^30^; and phosphatase and tensin homolog (PTEN)^31^, etc.

So far there is no literature data available on the cardiac expression of miR-212 and its targets in CKD. Therefore, we aimed to investigate the potential role of miR-212 and its hypertrophy-associated targets in LVH in CKD.

## Results

### The development of CKD in 5/6 nephrectomized rats

**Figure 1:**
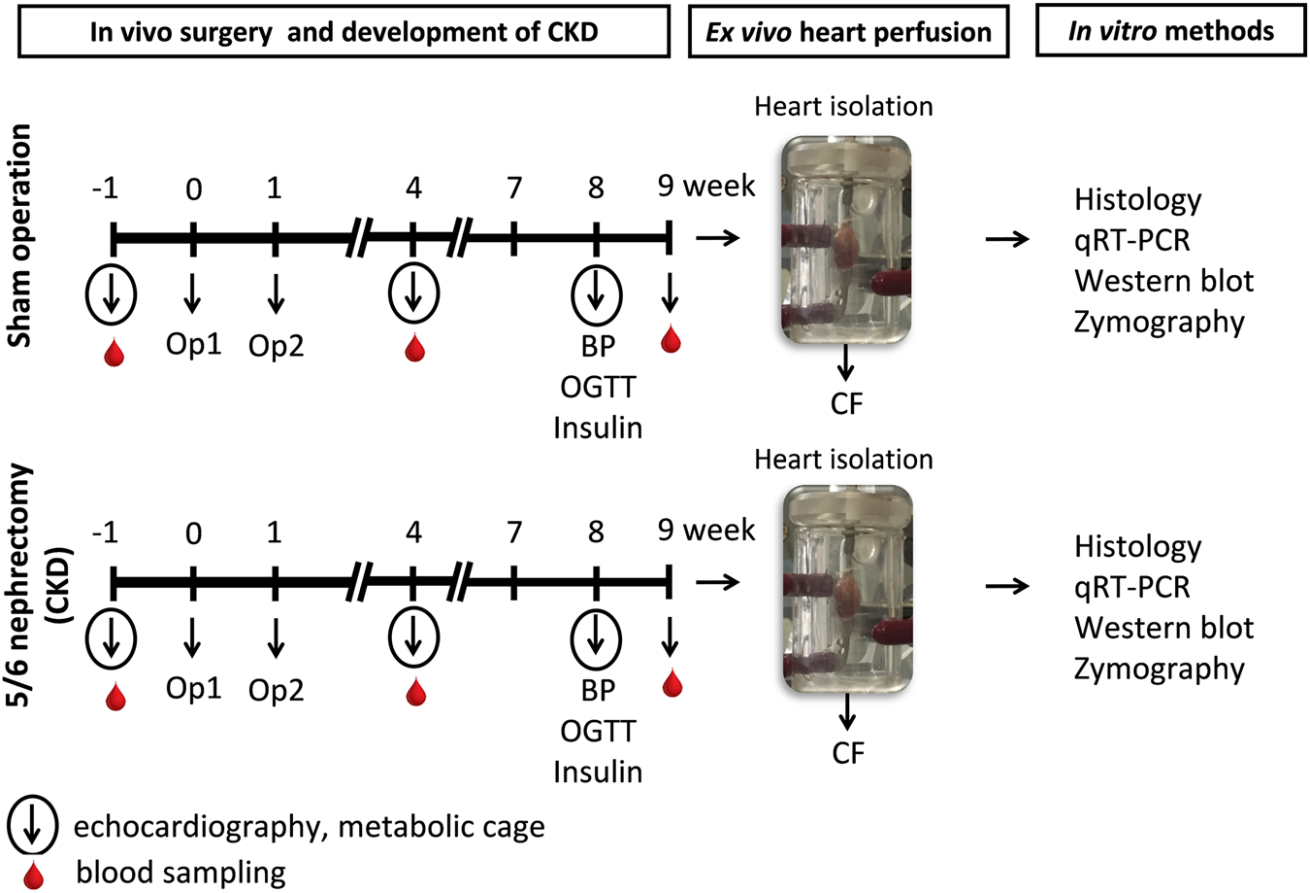
Experimental protocol. BP: blood pressure, CF: coronary flow, CKD: chronic kidney disease, OGTT: oral glucose tolerance test, Op: operation.

**Figure 2:**
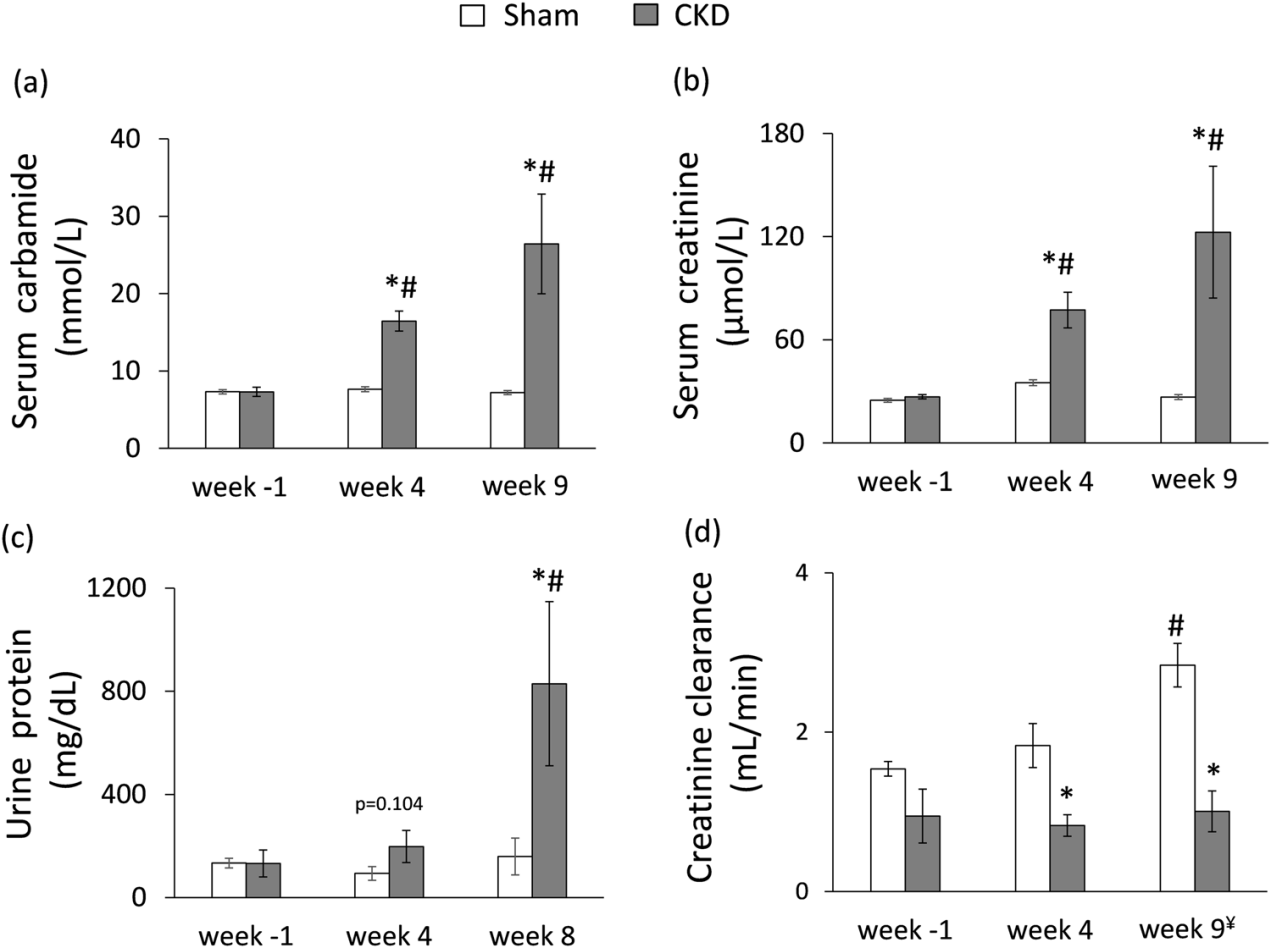
The development of chronic kidney disease. (**a**) Serum carbamide level, (**b**) serum creatinine level, (**c**) urine protein level (numeric p-value over the bars refers to a comparison between groups within the same time point), (**d**) creatinine clearance was calculated from urine volume, urine and serum creatinine concentrations according to a formula (urine creatinine concentration [μM] × urine volume for 24 h [mL])/(serum creatinine concentration [μM] × 24 × 60 min). ^¥^Urine volume and urine creatinine concentration were measured at week 8 and serum creatinine concentration was determined at week 9. White bars represent the sham-operated group and grey bars represent the chronic kidney disease (CKD) group. Values are means ± SEM, n = 9–10, *p < 0.05 vs. sham within the same time point, ^#^p < 0.05 vs. week −1.

During the follow-up period, the survival rate was 100% among sham-operated animals and 85% among 5/6 nephrectomized animals. Concentrations of serum and urine metabolites were measured at week −1, 4 and at the endpoint (week 8 in case of urine parameters and week 9 in case of serum parameters) to verify the development of CKD induced by 5/6 nephrectomy (Figs 1 and 2). The serum carbamide and creatinine levels were significantly increased at week 4 and the endpoint in the 5/6 nephrectomized rats as compared to the baseline values or the values of the sham-operated animals at each time point representing continously worsening renal function and the development of CKD (Fig. 2a,b). Likewise, in the 5/6 nephrectomized rats, urine protein concentration was significantly increased showing impaired glomerular function at the endpoint (Fig. 2c). Accordingly, creatinine clearance was significantly decreased in the 5/6 nephrectomized rats both at week 4 and at the endpoint as compared to the sham-operated rats showing the development of CKD (Fig. 2d). By contrast, creatinine clearance was significantly increased in the sham animals at the endpoint as compared to the baseline values due to normal growth of healthy animals (Fig. 2d). Serum potassium and magnesium ions were significantly increased at week 9 in the CKD group as compared to the sham group showing disturbed electrolyte homeostasis (Table 1). Glucose intolerance and insulin resistance are common complications in CKD and elevated insulin level could have a hypertrophic effect on the heart. Interestingly, there was no significant difference in blood glucose levels between the two groups at any time point of the oral glucose tolerance test (OGTT) at week 8 excluding glucose intolerance in the CKD group (Table 1). However, plasma insulin concentration was significantly lower in the CKD animals at week 8 as compared to sham-operated animals suggesting diminished function of the pancreatic beta cells (Fig. 1 and Table 1).

**Table 1:** Serum and plasma parameters in the CKD model.

Parameter (unit)	endpoint		
	Sham	CKD	p-value
Serum sodium ion (mmol/L)	144 ± 1	144 ± 1	0.786
Serum potassium ion (mmol/L)	5.50 ± 0.26	6.46 ± 0.34*	0.039
Serum chloride ion (mmol/L)	104 ± 1	102 ± 1	0.083
Serum calcium ion (mmol/L)	2.61 ± 0.02	2.66 ± 0.11	0.556
Serum magnesium ion (mmol/L)	0.95 ± 0.02	1.30 ± 0.14*	0.012
Serum phosphate ion (mmol/L)	2.55 ± 0.06	3.21 ± 0.45	0.117
Blood glucose 0′ min (mmol/L)	5.55 ± 0.13	5.48 ± 0.16	0.719
Blood glucose 30′ min (mmol/L)	7.24 ± 0.35	7.85 ± 0.34	0.235
Blood glucose 60′ min (mmol/L)	7.36 ± 0.24	7.96 ± 0.23	0.088
Blood glucose 90′ min (mmol/L)	5.71 ± 0.12	5.86 ± 0.17	0.519
Plasma insulin/plasma protein (ug/g)	0.032 ± 0.001	0.012 ± 0.002*	0.009

Values are mean ± SEM, n = 9–10, *p < 0.05 vs. control, unpaired t-test. CKD: chronic kidney disease.

### The development of HFpEF in CKD

**Figure 3:**
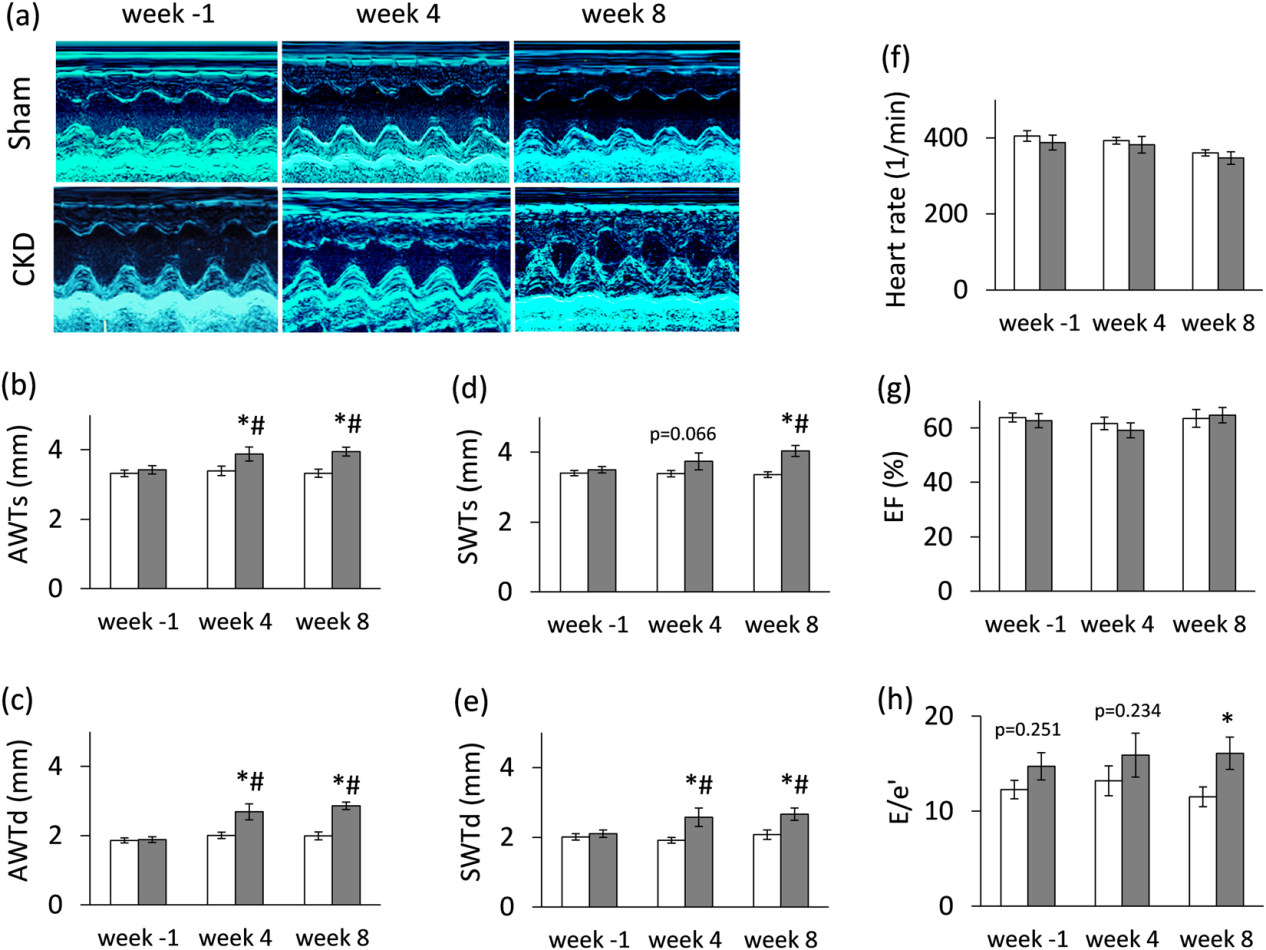
Echocardiographic results. (**a**) Representative M-mode images, (**b**) anterior wall thicknesses in systole (AWTs), (**c**) anterior wall thicknesses in diastole (AWTd), (**d**) septal wall thicknesses in systole (SWTs), (**e**) septal wall thicknesses in diastole (SWTd), (**f**) heart rate, (**g**) ejection fraction, (**h**) E/e′ ratio. White bars represent the sham-operated group, and grey bars represent the chronic kidney disease (CKD) group. Values are means ± SEM, n = 9–10, *p < 0.05 vs. sham within the same time point, ^#^p < 0.05 vs. week -1. (Numeric p-values over the bars refer to the comparison between groups within the same time point)

Transthoracic echocardiography was performed at week −1, 4 and 8 to investigate whether the development of CKD leads to an alteration in myocardial morphology and function (Figs 1, 3 and Table 2). At week −1, there was no difference in any measured parameters between the two groups (Fig. 3 and Table 2). At week 4, the diastolic function-associated e′ was significantly decreased as compared to the sham group (Table 2), and the anterior and septal diastolic wall thicknesses were significantly increased in the CKD group as compared to the sham group or baseline values showing starting LVH with mild diastolic dysfunction (Fig. 3). At week 8, left ventricular wall thicknesses including anterior and septal walls both in systole and diastole were significantly increased in CKD rats as compared to the sham group and the baseline values pointing to the presence of LVH (Fig. 3a–e). The inferior and posterior wall thicknesses in diastole showed a tendency of increase in the CKD group as compared to the sham group at week 8 (Table 2). Both end-systolic and end-diastolic diameters were significantly increased in both groups as compared to baseline values at week 4 and 8 due to the growth of the animals (Table 2). Left ventricular end-systolic, end-diastolic, and stroke volumes were also decreased in the CKD group as compared to the sham group at week 8 (Table 2). Ejection fraction remained unchanged in the CKD rats compared to the sham rats or the baseline values both at week 4 and 8 showing a characteristic picture of the entity called HFpEF (Fig. 3g). There was no significant difference in heart rate between the two groups at any time point (Fig. 3f). Cardiac output was significantly reduced in the CKD rats as compared to the sham group at week 8 (Table 2). More importantly, the ratio of the early flow velocity E and the septal mitral annulus velocity e′ significantly increased in CKD rats at week 8 indicating diastolic dysfunction (Fig. 3h). Another parameter of the diastolic function, the isovolumic relaxation time was significantly longer in the CKD animals as compared to controls at week 8 pointing to abnormal myocardial relaxation (Table 2). Systolic parameters including isovolumic contraction time and ejection time and the myocardial performance index (Tei index) remained unchanged in the CKD group as compared to the sham group at any time point (Table 2). Hypertension is a well-known complication in CKD and also an independent risk factor for the development of LVH. Interestingly, there was only a tendency of a slight increase in blood pressure in the CKD group as compared to the sham-operated group at week 8 (Table 3).

**Table 2:** Effects of CKD on various *in vivo* left ventricular morphological and functional parameters measured by transthoracic echocardiography.

	Parameter (unit)	View/Mode	week −1			week 4			week 8		
			Sham	CKD	p-value	Sham	CKD	p-value	Sham	CKD	p-value
**LV MORPHOLOGY**	Inferior wall thickness-systolic (mm)	short axis/MM	3.24 ± 0.14	3.12 ± 0.11	0.553	2.97 ± 0.19	3.18 ± 0.17	0.416	3.16 ± 0.16	3.41 ± 0.24	0.381
	Inferior wall thickness-diastolic (mm)	short axis/MM	2.00 ± 0.23	1.59 ± 0.07	0.132	1.83 ± 0.12	2.01 ± 0.14	0.325	1.87 ± 0.12	2.39 ± 0.26^#^	0.081
	Posterior wall thickness-systolic (mm)	long axis/MM	3.51 ± 0.14	3.33 ± 0.16	0.394	3.07 ± 0.14	3.48 ± 0.20	0.107	3.13 ± 0.16	3.44 ± 0.27	0.305
	Posterior wall thickness-diastolic (mm)	long axis/MM	1.97 ± 0.10	1.91 ± 0.15	0.773	1.75 ± 0.11	2.23 ± 0.22	0.057	1.76 ± 0.10	2.27 ± 0.29	0.099
	Left ventricular end diastolic diameter (mm)	long axis/MM	4.81 ± 0.21	4.68 ± 0.28	0.699	6.25 ± 0.24^#^	5.88 ± 0.38^#^	0.400	6.58 ± 0.25^#^	6.40 ± 0.43^#^	0.715
	Left ventricular end diastolic diameter (mm)	short axis/MM	4.55 ± 0.37	5.12 ± 0.27	0.243	6.02 ± 0.15^#^	6.22 ± 0.33^#^	0.562	6.40 ± 0.26^#^	6.22 ± 0.40^#^	0.712
	Left ventricular end systolic diameter (mm)	long axis/MM	1.44 ± 0.18	1.89 ± 0.29	0.190	2.59 ± 0.27^#^	2.48 ± 0.26	0.779	3.32 ± 0.25^#^	2.97 ± 0.39^#^	0.453
	Left ventricular end systolic diameter (mm)	short axis/MM	1.85 ± 0.45	1.44 ± 0.26	0.462	2.89 ± 0.23^#^	2.79 ± 0.33^#^	0.790	3.03 ± 0.21^#^	3.17 ± 0.44^#^	0.769
**LV FUNCTION**	Fractional shortening (%)	long axis/MM	70 ± 3	66 ± 3	0.297	59 ± 4^#^	58 ± 4^#^	0.797	50 ± 2^#^	55 ± 4^#^	0.247
	Fractional shortening (%)	short axis/MM	71 ± 3	73 ± 3	0.683	56 ± 3^#^	57 ± 4^#^	0.860	53 ± 2^#^	51 ± 5^#^	0.745
	Left ventricular end-diastolic volume (µl)	4CH/2D	71 ± 10	74 ± 12	0.826	87 ± 12	122 ± 20^#^	0.145	136 ± 20^#^	78 ± 7*	0.014
	Left ventricular end-systolic volume (µl)	4CH/2D	27 ± 4	27 ± 3	0.960	34 ± 6	52 ± 12^#^	0.198	55 ± 12^#^	27 ± 3	0.054
	Stroke volume (µl)	4CH/2D	45 ± 6	47 ± 7	0.756	53 ± 6	70 ± 9	0.141	82 ± 8^#^	51 ± 5*	0.004
	Cardiac output (mL/min)	4CH/2D	18 ± 3	19 ± 3	0.932	21 ± 2	26 ± 4	0.225	29 ± 2^#^	17 ± 2*	0.001
	E-wave (m/s)	4CH/PWD	0.84 ± 0.07	0.84 ± 0.06	0.961	0.99 ± 0.08	0.88 ± 0.07	0.317	0.71 ± 0.03	0.73 ± 0.06	0.718
	e′-wave (m/s)	4CH/TD	0.065 ±0.003	0.059 ±0.003	0.217	0.079 ± 0.003	0.063 ± 0.008*	0.027	0.066 ± 0.005	0.049 ± 0.005*	0.014
	Isovolumic contraction time (ms)	4CH/PWD	17.17 ±1.38	18.26 ± 1.92	0.644	14.37 ± 1.10	16.85 ± 0.56	0.069	17.53 ± 1.19	20.19 ± 1.80	0.227
	Isovolumic relaxation time (ms)	4CH/PWD	27.20 ± 2.72	26.37 ± 3.04	0.840	22.83 ± 2.01	26.52 ± 2.32	0.244	25.50 ± 1.71	32.33 ± 2.68*	0.042
	Ejection time (ms)	4CH/PWD	50.60 ± 2.17	45.22 ± 4.29	0.264	54.17 ± 2.37	61.00 ± 5.88^#^	0.278	62.13 ± 2.10	66.15 ± 3.54^#^	0.331
	Tei index	4CH/PWD	0.90 ± 0.09	1.01 ± 0.08	0.264	0.71 ± 0.07	0.76 ± 0.07	0.574	0.71 ± 0.06	0.75 ± 0.06	0.665

Transthoracic echocardiographic measurements were performed −1, 4, and 8 weeks after 5/6 nephrectomy. Values are mean ± SEM, n = 9–10, *p < 0.05 vs. control, unpaired t-test within each time point. p-values refer to the unpaired t-test at each time point. ^#^p < 0.05 vs^.^ week −1, repeated measures two-way ANOVA (Holm-Sidak post hoc test). 2D, two dimensional, 4CH: four chambers view, CKD: chronic kidney disease; E-wave, early ventricular filling velocity; MM, M (motion) Mode; PWD, pulse wave Doppler; TD, tissue Doppler.

**Table 3:** The effect of CKD on blood pressure, body weight and heart weight.

Parameters	Sham	CKD	p-value
Body weight (g) (week −1)	283 ± 6	284 ± 12	0.954
Body weight (g) (week 9)	431 ± 13	425 ± 13	0.734
Heart weight (mg)	1193 ± 41	1318 ± 55*	0.031
Heart weight/body weight (mg/g)	2.77 ± 0.06	3.11 ± 0.13*	0.029
Systolic blood pressure (mmHg)	138 ± 3	146 ± 5	0.145
Diastolic blood pressure (mmHg)	117 ± 2	122 ± 4	0.240
Mean arterial blood pressure (mmHg)	127 ± 2	134 ± 4	0.148

Values are mean ± SEM, n = 9–10, *p < 0.05 vs. control, unpaired t-test. CKD: chronic kidney disease. Blood pressure was measured at week 8 in a subgroup of animals. Heart weight was measured at week 9.

At week 9, at autopsy, heart weight and heart weight to body weight ratio were significantly increased in CKD animals than in controls indicating macroscopic signs of hypertrophy (Table 3). Moreover, basal coronary flow – measured in isolated perfused hearts - was significantly reduced in the CKD group as compared to the sham group (13 ± 0.5 vs. 18 ± 1.5 mL/min) supporting the reduced cardiac output assessed by echocardiography *in vivo* (Table 2). At autopsy, the weight of the whole left kidney in the sham-operated group was smaller than the remaining one-third of the left kidney in the CKD group (1145 ± 75 vs. 2053 ± 118 mg) suggesting marked renal hypertrophy in the CKD animals.

### Cardiomyocyte hypertrophy and interstitial fibrosis in CKD

**Figure 4:**
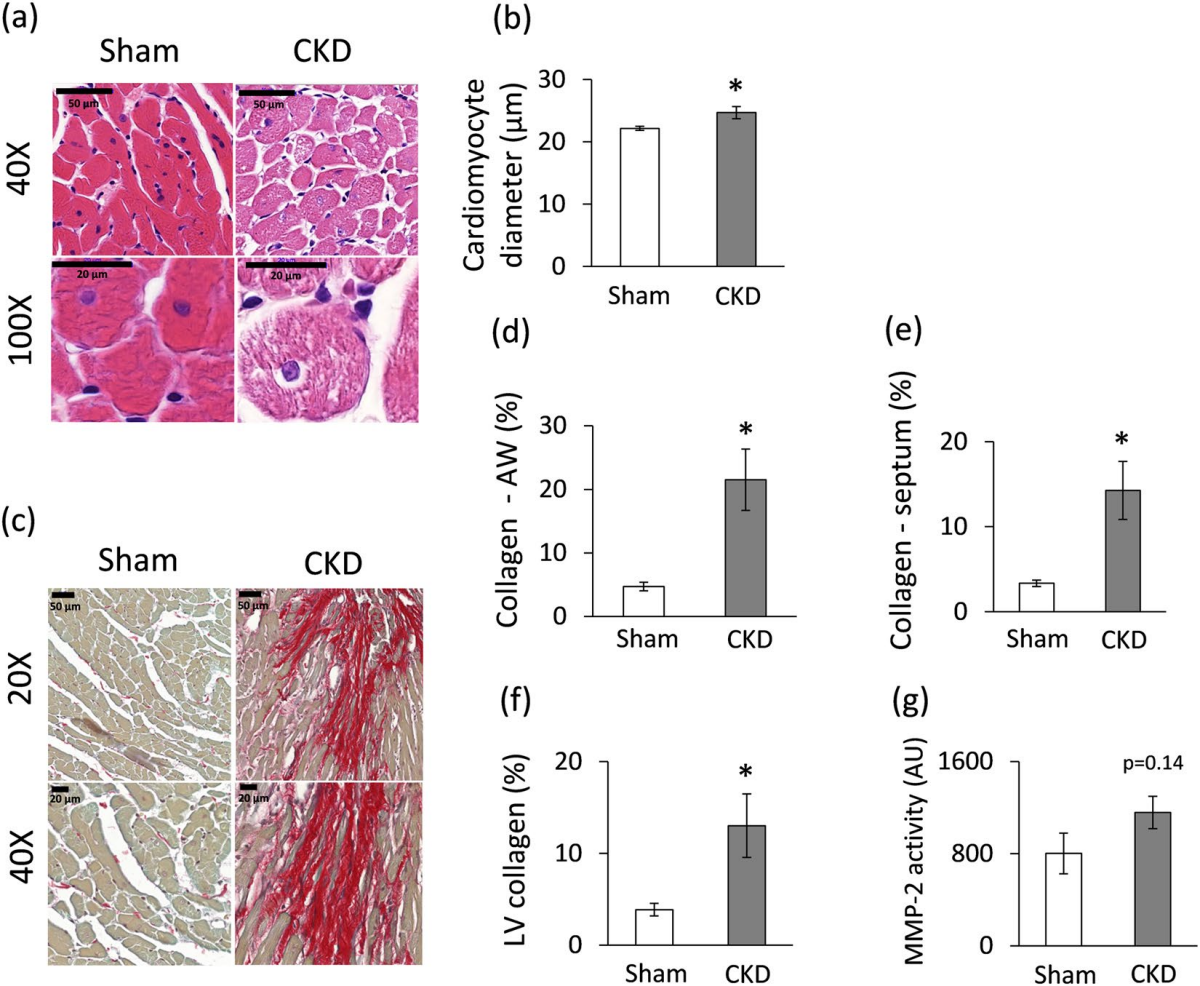
Histology and zymography results at week 9. (**a**) Hematoxylin-eosin stained slides, black scale bars represent 50 µm and 20 µm at 40X and 100X magnification, respectively, (**b**) Cardiomyocyte diameter, (**c**) Picrosirius red and fast green stained slides, black scale bars represent 50 µm and 20 µm at 20X and 40X magnification, respectively, (**d**) Anterior wall (AW) collagen content, (**e**) Septal collagen content (**f**) Left ventricular (LV) collagen content, (**g**) MMP-2 activity. White bars represent the sham-operated group, and grey bars represent the chronic kidney disease (CKD) group. Values are means ± SEM, n = 8–9, *p < 0.05, unpaired t-test.

Cardiomyocyte diameters were measured histologically to verify the development of LVH seen on echocardiographic images and at autopsy (Fig. 4a,b). Cross-sectional cardiomyocyte diameters were significantly increased in the CKD group as compared to the sham group proving the presence of LVH at the cellular level (Fig. 4a,b).

Collagen deposition was assessed to investigate the development of fibrosis in CKD (Figs. 4c–f). Significant interstitial fibrosis was found with consistent interstitial collagen depositions in all studied segments of the CKD hearts (Fig. 4c–f). Matrix metalloprotease 2 (MMP-2) can break down collagen in the extracellular matrix. Its cardiac activity showed a tendency of increase in CKD as compared to the sham group (Fig. 4g) suggesting that the balance between collagen breakdown and deposition might have been shifted towards deposition.

### Molecular markers of LVH and heart failure in CKD

The expression of LVH- and heart failure-associated markers were measured by qRT-PCR. Among hypertrophy markers, left ventricular expression of alpha-MHC (*Myh6*) was significantly decreased in CKD as compared to controls (Table 4). However, left ventricular mRNA level of beta-MHC (*Myh7*) did not change in CKD as compared to the sham group (Table 4). The increase in the beta-MHC to alpha-MHC ratio is a characteristic change in LVH. The left ventricular expression of the pro-hypertrophic myocyte enhancer factor 2 C (*Mef2c*) and myocyte enhancer factor 2D (*Mef2d*) did not increase in the CKD group as compared to the sham group (Table 4).

**Table 4:** The effect of CKD on the molecular markers of left ventricular hypertrophy and fibrosis.

Gene name	Gene symbol	log_2_ change	SD log_2_ change	p-value	Fold change
Natriuretic peptide A (ANP)	*Nppa*	1.80	1.88	0.000	3.48*
Natriuretic peptide B (BNP)	*Nppb*	−0.55	1.63	0.118	−1.46
Myosin, Heavy Polypeptide 6, Cardiac Muscle, Alpha (α-MHC)	*Myh6*	−1.26	1.34	0.000	−2.39*
Myosin, Heavy Polypeptide 7, Cardiac Muscle, Beta (β-MHC)	*Myh7*	−0.71	1.06	0.000	−1.64
Myocyte enhancer factor 2 C (predicted)	*Mef2c*	0.23	0.25	0.000	1.17
Myocyte enhancer factor 2D	*Mef2d*	0.05	0.21	0.201	1.03

Gene expression ratios (CKD vs. control). Fold change of <−1.75 or >1.75 (repression or overexpression, respectively) and a p-value of <0.05 were considered as a significant change*, n = 6, unpaired t-test or Mann Whitney U test (*Nppa*, *Nppb*, *and Mef2c*).

Among heart failure markers, the mRNA level of the A-type natriuretic peptide (ANP, [*Nppa*]) significantly increased in CKD as compared to the sham group, while the mRNA level of the B-type natriuretic peptide (BNP, [*Nppb*]) did not change significantly (Table 4).

### Left ventricular overexpression of miR-212 in CKD

**Figure 5:**
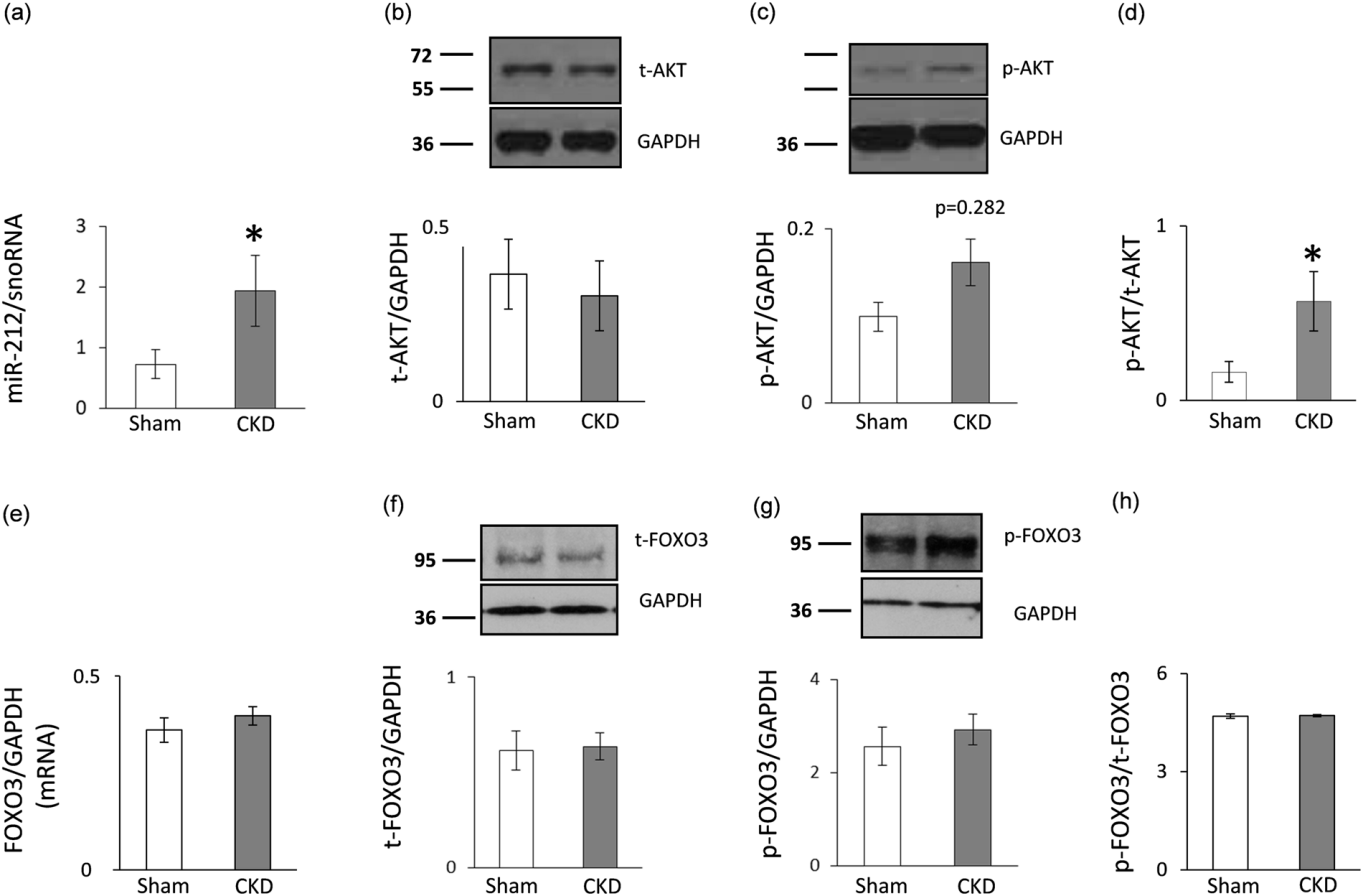
qRT-PCR and Western blot results at week 9. Left ventricular (**a**) miR-212 expression, (**b**) total AKT expression, (**c**) phospho-AKT expression, (**d**) phospho-AKT/total AKT ratio, (**e**) FOXO3 mRNA expression, (**f**) total FOXO3 expression, (**g**) phospho-FOXO3 expression, and (**h**) phospho-FOXO3/total FOXO3 ratio. White bars represent the sham-operated group, and grey bars represent the chronic kidney disease (CKD) group. Values are means ± SEM, n = 7–8, *p < 0.05, unpaired t-test (a–e) or Mann-Whitney-U test (f–h).

Left ventricular expression of miR-212 significantly increased in CKD as compared to the sham group (Fig. 5a).

### No change in left ventricular FOXO3 expression and phosphorylation in CKD

We measured the left ventricular expression of FOXO3 which is a validated target of miR-212^23,32^. Left ventricular FOXO3 level failed to decrease at the mRNA and the protein level in the CKD group as compared to the sham group (Fig. 5e–f). In pressure-overload-induced LVH, increased phospho-FOXO3/total FOXO3 ratio is a characteristic shift. However, in our current study, the left ventricular phospho-FOXO3 level and the phospho-FOXO3/total FOXO3 ratio was not increased in CKD as compared to the sham group (Fig. 5g–h).

### Increased left ventricular phospho-AKT/total AKT ratio in CKD

Phospho-AKT has been reported to play a role in the development of LVH both in a FOXO3-dependent or -independent manner in non-CKD-induced forms of LVH^32–34^. In our present study, left ventricular expression of total AKT did not differ between the two groups at the protein level (Fig. 5b). However, the expression of the phospho-AKT showed an increasing tendency in CKD hearts as compared to controls (Fig. 5c). The phospho-AKT/total AKT ratio significantly increased in CKD as compared to the sham group (Fig. 5d).

### No change in left ventricular mTOR expression and phosphorylation in CKD

**Figure 6:**
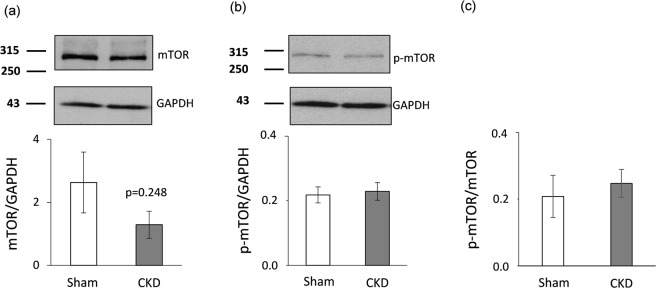
mTOR Western blot results at week 9. Left ventricular (**a**) total mTOR expression, (**b**) phospho-mTOR expression, and (**c**) phospho-mTOR/total mTOR ratio. White bars represent the sham-operated group, and grey bars represent the chronic kidney disease (CKD) group. Values are means ± SEM, n = 7–8, *p < 0.05, unpaired t-test.

The AKT/mTOR pathway has been suggested to be another crucial regulator of pressure-overload-induced LVH^33^. Therefore, the left ventricular expression of mTOR and phosho-mTOR were also measured at the protein level in our present study. The expression of both mTOR and phospho-mTOR failed to change significantly at the protein level in the CKD group as compared to the control group (Fig. 6a,b). Accordingly, phospho-mTOR/mTOR ratio did not change significantly in response to CKD (Fig. 6c).

### Molecular markers of the pro-hypertrophic calcineurin/NFAT pathway

Beyond other molecular pathways, FOXO3 protein regulates the pro-hypertrophic calcineurin/NFAT pathway^35^. This pathway was shown to be regulated by miR-212 via FOXO3 in LVH induced by pressure-overload^23^. In our present study, left ventricular expression of the marker molecules of the calcineurin/NFAT pathway was measured by qRT-PCR. The expression of protein phosphatase 3, catalytic subunit alpha and beta (i.e., calcineurin A-alpha, [*Ppp3ca*] and calcineurin A-beta, [*Ppp3cb*]), myocyte-enriched calcineurin-interacting protein 1 (MCIP1.4, [*Rcan1*]), and the calcineurin-dependent cytoplasmic nuclear factor of activated T-cells (*Nfatc4*) did not change in CKD as compared to the sham group (Table 5). In contrast, the atrophic gene atrogin-1 (*Fbx32*) was significantly down-regulated in CKD as compared to the sham group (Table 5).

**Table 5:** The effect of CKD on molecular markers of the calcineurin/NFAT pathway at the mRNA level.

Gene name	Gene symbol	log_2_ change	SD log_2_ change	p-value	Fold change
Protein phosphatase 3 catalytic subunit alpha	*Ppp3ca*	0.14	0.21	0.000	1.10
Protein phosphatase 3 catalytic subunit beta	*Ppp3cb*	0.01	0.38	0.896	1.01
Nuclear Factor Of Activated T-Cells, Cytoplasmic, Calcineurin-Dependent 4	*Nfatc4*	−0.05	0.97	0.775	−1.03
Muscle Atrophy F-Box Protein (atrogin-1)	*Fbx32*	−0.92	1.21	0.001	−1.89
Myocyte-Enriched Calcineurin-Interacting Protein 1 (MCIP1.4)	*Rcan1*	−0.12	1.81	0.781	−1.09

Gene expression ratios (CKD vs. control). Fold change of <−1.75 or >1.75 (repression or overexpression, respectively) and a p-value of <0.05 were considered as a significant change, n = 5–6, unpaired t-test.

### Hypertrophy-associated targets of miR-212 beyond FOXO3: no change in left ventricular ERK1/2 and AMPK expression and phosphorylation in CKD

**Figure 7:**
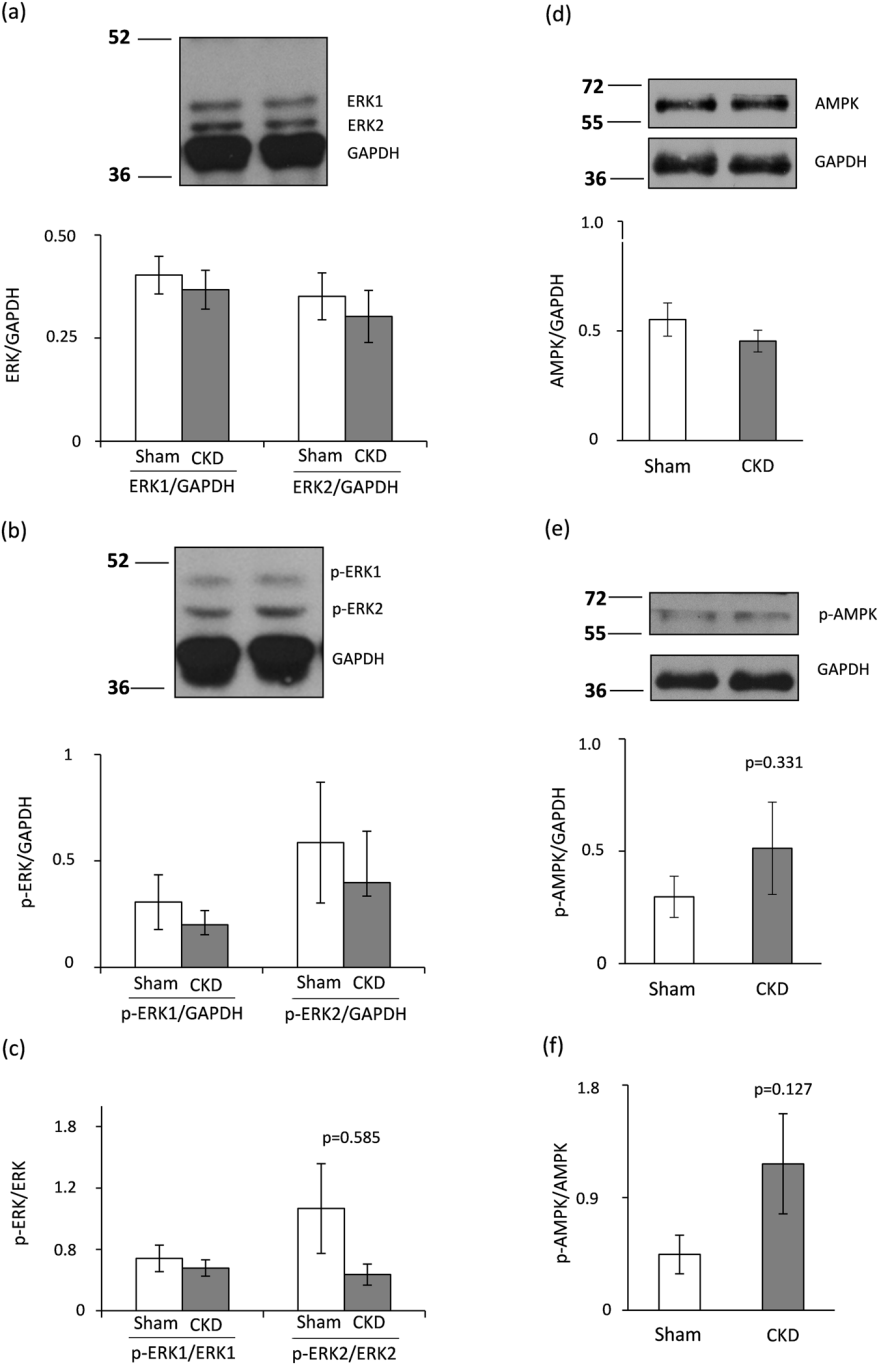
ERK1/2 and AMPK Western blot results at week 9. Left ventricular (**a**) total ERK1 and ERK2 expressions, (**b**) phospho-ERK1 and phospho-ERK2 expressions, (**c**) phospho-ERK1/total ERK1 and phospho-ERK2/total ERK2 ratios, (**d**) total AMPK expression, (**e**) phospho-AMPK expression, and (**f**) phospho-AMPK/total AMPK ratio. White bars represent the sham-operated group, and grey bars represent the chronic kidney disease (CKD) group. Values are means ± SEM, n = 7–8, *p < 0.05, unpaired t-test.

Beyond FOXO3, several hypertrophy-associated predicted targets of miR-212 were also measured in the current study. We have investigated the left ventricular expression of the extracellular signal-regulated kinase 2 (ERK2, [*Mapk1*]), the myocyte enhancer factor 2a (*Mef2a*); the protein kinase AMP-activated catalytic subunit alpha 2 (AMPK, [*Prkaa2*]); DnaJ heat shock protein family member A2 (HSP40, [*Dnaja2*]); sirtuin 1, transcript variant X1 (*Sirt1*); and phosphatase and tensin homolog (*Pten*) at the transcript level (Table 6). These target mRNAs failed to show significant down-regulation in response to CKD as compared to control hearts (Table 6). We selected ERK2 (measured together with ERK1) and AMPK to determine their cardiac expression also at the protein level (Fig. 7). In our current study, left ventricular total ERK1 and ERK2 levels, phospho-ERK1 and phospho-ERK2 levels, phospho-ERK1/total ERK1 ratio, phospho-ERK2/total ERK2 ratio, total AMPK level, phospho-AMPK level, and phospho-AMPK/total AMPK ratio failed to change significantly in CKD as compared to the sham group (Fig. 7). Therefore, these targets of miR-212 do not seem to play a crucial role in the development of LVH in CKD.

**Table 6:** The effect of CKD on hypertrophy-associated selected targets of miR-212.

Gene name	Gene symbol	log_2_ change	SD log_2_ change	p-value	Fold change
Mitogen activated protein kinase 1 (ERK2)	*Mapk1*	−0.22	0.24	0.000	−1.17
Myocyte enhancer factor 2a	*Mef2a*	−0.11	0.29	0.026	−1.08
Protein kinase AMP-activated catalytic subunit alpha 2 (AMPK)	*Prkaa2*	−0.22	0.34	0.000	−1.16
DnaJ heat shock protein family (HSP40) member A2	*Dnaja2*	−0.18	0.18	0.002	0.88
Sirtuin 1, transcript variant X1 (predicted)	*Sirt1*	−0.02	0.34	0.750	−1.01
Phosphatase and tensin homolog	*Pten*	0.13	0.25	0.003	1.10

Gene expression ratios (CKD vs. control). Fold change of <−1.75 or >1.75 (repression or overexpression, respectively) and a p-value of <0.05 were considered as a significant change*, n = 5–6, unpaired t-test or Mann Whitney U test (*Dnaja2*).

## Discussion

5/6 nephrectomy is probably the most established model of progressive renal failure with loss of renal mass^36^. 5/6 nephrectomy mimics the consequences of the reduction of functional nephron number^36^. In this model of CKD, cardiac hypertrophy, interstitial fibrosis, and diastolic dysfunction are consistent characteristics; however, severe hypertension is not usually a feature of this model^36–39^. In our current study, the development of CKD, and significant cardiac morphological and functional changes were confirmed 8 and/or 9 weeks after 5/6 nephrectomy. Animals presented LVH and diastolic dysfunction which are the hallmarks of HFpEF. Cardiac interstitial fibrosis was also present in our CKD model. Since interstitial fibrosis is a final major endpoint of cardiac pathologies in CKD, our model also seems to be an adequate one in a series of future investigations with different anti-fibrotic agents. In our present model, interstitial fibrosis and cardiomyocyte hypertrophy might lead to increased cardiac stiffness and diastolic dysfunction without hypertension. The lack of severe hypertension, atherosclerosis and glucose intolerance makes it possible to study the direct effects and mechanisms of CKD on left ventricular hypertrophy and fibrosis. While traditional cardiovascular risk factors such as hypertension, atherosclerosis, and diabetes mellitus, are highly prevalent in CKD patients, results of clinical trials focusing on controlling such factors have been largely disappointing^36^. Both pre-clinical and clinical studies proved that factors related to CKD itself provoke the development of LVH and fibrosis, regardless of pressure- and volume-overload^13–17^. The CKD-specific risk factors can contribute to the development of cardiac hypertrophy and remodeling. These factors may include (i) uremic toxins (such as indoxyl sulfate) inducing pro-fibrotic and oxidative pathways^36–40^, (ii) renal anemia due to erythropoietin deficiency^36,41^, (iii) neurohormonal disturbances including the activation of the renin-angiotensin-aldosterone system and sympathetic nervous system with decreased nitric-oxide levels^36,42^, (iv) secondary hyperparathyroidism with hyperphosphatemia^36,43^, (v) fibroblast growth factor 23 (FGF23), primarily involved in CKD-induced mineral and bone disorder^36,44^, (vi) insulin resistance and glucose intolerance leading to cardiometabolic complications^36,45^, *etc*.

MiRs have been shown to be crucial contributors to cardiovascular biology and disease development^19–24,46^. So far, only a few studies have been published describing the role of miRs in the development of LVH and fibrosis in CKD. Chuppa *et al*. published recently that the suppression of miR-21 protected rats with 5/6 nephrectomy from developing LVH and improved left ventricular function via PPAR-alpha-mediated pathways^47^. It has also been reported that activated Na^+^/K^+^-ATPase signaling could repress the cardiac expression of miR-29b which lead to increased collagen synthesis in CKD^48^. Moreover, repression of miR-29b and miR-30c played a role in the development of cardiac fibrosis in CKD via targeting the pro-fibrotic molecules collagen-1a1, matrix metalloproteinase 2 and connective tissue growth factor^49^. Cardiac repression of miR-208 and overexpressed circulating miR-133a were also associated with cardiac hypertrophy in CKD^50,51^. However, there is no literature data on the cardiac expression of the pro-hypertrophic miR-212 in CKD so far. It has been previously demonstrated that miR-212 is overexpressed in human heart failure^24^ and transverse aortic constriction-induced LVH and heart failure in mice^23^. MiR-212 has been shown to be a key regulator in the development of LVH and heart failure via the repression of the anti-hypertrophic transcription factor FOXO3 and the overactivation of the calcineurin/NFAT signaling during heart failure development^23^. In our present study, LVH and HFpEF in CKD were accompanied by the overexpression of miR-212. By contrast, the cardiac expression of FOXO3 failed to decrease at the mRNA and the protein level in CKD. Moreover, the phospho-FOXO3/total FOXO3 ratio did not change in CKD. Therefore, our results suggest that FOXO3 might not play a substantial role in the development of LVH and fibrosis in CKD.

The protein kinase AKT was also reported to be a crucial regulator of LVH induced by pressure-overload^32–34^. AKT could be regulated via both FOXO3-dependent and independent pathways^33–35^. Therefore, we measured the protein level of both total AKT and phospho-AKT. In our present study, the phospho-AKT/total AKT ratio significantly increased which is a characteristic shift in pressure-overload-induced cardiac hypertrophy^52^. Moreover, the AKT/mTOR pathway plays a crucial role in the development of left ventricular hypertrophy^33,53^. Therefore, the expression of mTOR and phospho-mTOR proteins were measured and no difference was found between the sham-operated and CKD group in our present study. In contrast, a preclinical study reported that 12 months long treatment with the mTOR inhibitor rapamycin resulted in the normalization of heart weight and cardiac mTOR signaling pathway in Han:SPRD rats with polycystic kidney disease^54^. In our present study, we used a model in the early stages of CKD with preserved systolic function. The different duration and severity of our CKD model as compared to the aforementioned Han:SPRD rats^54^ might explain the different mTOR expression in our present study.

The hypertrophic calcineurin/NFAT pathway could also be activated via FOXO3-dependent and independent mechanisms^52,55^. NFAT proteins are dephosphorylated by the Ca^2+^∕calmodulin-dependent phosphatase calcineurin and translocated to the nucleus to activate target gene expression^56^. In our present study, cardiac mRNA levels of calcineurin A-alpha, calcineurin A-beta, and NFAT did not change in the CKD hearts as compared to controls suggesting that calcineurin and NFAT are not overexpressed in LVH in CKD. Moreover, overexpressed FOXO3 was reported to increase the expression of the atrophic gene atrogin-1 at the mRNA level, and atrogin-1 could directly reduce the activity of calcineurin-A^57^. In our present study, FOXO3 failed to change, and atrogin-1 was significantly repressed at the mRNA level in CKD. The cardiac expression of atrogin-1 might be regulated by FOXO3-independent mechanisms in CKD (e.g., miRs beyond miR-212).

To find potential FOXO3-independent pro-hypertrophic mechanisms in CKD, we also investigated the expression of several other regulatory molecules that are predicted targets of miR-212 and associated with LVH. Target mRNAs were myocyte enhancer factor 2a (*Mef2a*); AMP-activated protein kinase (AMPK, [*Prkaa2*]); heat shock protein 40 (HSP40, [*Dnaja2*]); sirtuin 1 (*Sirt1*); phosphatase and tensin homolog (*Pten*), extracellular signal-regulated kinase 2 (ERK2, [*Mapk1*]). These target mRNAs failed to show significant repression in the cardiac tissue samples of CKD animals as compared to control hearts. We further investigated the cardiac expression of ERK1, ERK2 and AMPK at the protein level in our present study. In our hands, no significant change was found in the cardiac expression and phosphorylation of ERK1, ERK2 and AMPK in CKD as compared to the sham-operated animals. Literature data are controversial on the cardiac activity of ERK1/2 pathway in CKD. Liu *et al*. showed increased cardiac phospho-ERK/total ERK ratio in male Sprague Dawley rats 8 weeks after 5/6 nephrectomy^58^. In contrast, Hernandez-Resendiz *et al*. found decreased phospho-ERK/total ERK ratio in male Wistar rats 4 weeks after 5/6 nephrectomy^59^. Haq et *al*. reported that ERK1/2 and phosphoinositide 3-kinase/ protein kinase B/glycogen synthase kinase-3β (PI3K/AKT/GSK-3β) cascades are not the predominant pathways in human hearts with compensated LVH^60^. However, this pattern is reverted in failing hearts^60^. Therefore, a plausible explanation for our findings may be that our CKD model represents the compensated phase of LVH. Interestingly, AMPK was implicated as a central regulator of the development of LVH induced by pressure-overload either directly or indirectly via numerous downstream pathways including, e.g., mTOR, FOXO3, etc.^61^. Literature data are very limited on the cardiac expression of AMPK in CKD. Yang *et al*. reported that a uremic toxin indoxyl sulfate induced hypertrophy by inhibiting the AMPK/uncoupling protein 2 signaling pathway in neonatal rat cardiomyocytes^62^. However, there is no data available on cardiac AMPK expression at the protein level in animal studies in CKD.

The development of the HFpEF in CKD seems to be unique and distinct from other types of cardiac hypertrophies, such as the commonly studied compensatory hypertrophy and subsequent heart failure type induced by pressure-overload. In CKD, distinct mechanisms, e.g., unique CKD-associated factors, compensatory hypertrophy, pathologic remodeling, cell death, and survival mechanisms could be activated simultaneously. The individual pathways may converge toward key down-stream regulators (e.g., FOXO3, AKT, ERK1/2, AMPK, mTOR, etc.) and the ensuing effects may cancel each other, as reflected in unchanged protein levels.

In summary, this is the first study to report that LVH and fibrosis in CKD are accompanied by characteristic miR-212 overexpression in the left ventricle. However, it needs to be further explored whether the cardiac overexpression of miR-212 is a cause or consequence of LVH in CKD. The molecular mechanism of the development of LVH and fibrosis in CKD seems to be distinct from other forms of hypertrophy and pathological remodeling.

## Limitations

Our results regarding altered cardiac gene expression due to CKD are based on selected miRs and target molecules, however, measurement of the full rat transcriptome should be performed in the future. In this study, the focus was on miR-212 with its selected hypertrophy-associated target molecules, the relative role of other miRs or other miR-212 targets were not assessed in the development of cardiac hypertrophy in CKD. This study is descriptive; therefore future studies providing more in-depth mechanistic insight are necessary. Moreover, this is an exploratory study, thus therapeutic intervention was out of the scope.

## Materials and Methods

This investigation conformed to the National Institutes of Health Guide for the Care and Use of Laboratory Animals (NIH Publication No. 85-23, Revised 1996) and was approved by the Animal Research Ethics Committee of Csongrád County (XV.1181/2013) and the University of Szeged (MÁB-I-74-2017) in Hungary. All institutional and national guidelines for the care and use of laboratory animals were followed.

### Animals

A total of 66 adult male Wistar rats (250–300 g) were used in this study. 30 animals underwent sham-operation, and 36 animals received 5/6 nephrectomy to induce CKD. Animals were housed in pairs in individually ventilated cages (Sealsafe IVC system, Italy) and were maintained in a temperature-controlled room with a 12-h:12-h light/dark cycles throughout the study. Standard rat chow and tap water were supplied *ad libitum*.

### Experimental setup

Experimental CKD was induced by 5/6 nephrectomy. Animals underwent a sham operation or 5/6 nephrectomy in two phases as we described previously^63^ (Fig. 1). After the operations, both groups were followed-up for 9 weeks. At week −1, 4 and 8, cardiac morphology and function were assessed by transthoracic echocardiography in a subgroup of both the 5/6 nephrectomized and sham-operated rats (n = 10) (Fig. 1). At week −1, and 4, blood was collected from vena saphena (n = 10), and at week 9 from the thoracic aorta (n = 9–10), and serum urea and creatinine levels were measured. Serum ion levels were determined only at week 9. Moreover, this subgroup of animals was placed in metabolic cages at week −1, 4 and 8 for 24 h to measure urine creatinine and protein levels (Fig. 1). At week 8, oral glucose tolerance test (OGTT), blood glucose, and plasma insulin measurement were also performed in another subgroup of animals (n = 9–10). In a separated subgroup of animals, invasive blood pressure measurements were performed in the right carotid artery at week 8 (Fig. 1) (n = 10–13). At the termination of the experiment at week 9, rats were anesthetized, and hearts were isolated and perfused according to Langendorff, then left ventricles were separated, and samples were prepared for histology and biochemical measurement. The development of LVH and fibrosis in CKD was verified by the measurement of myocardial fibre diameters as well as picrosirius red staining for collagen. Total RNA was isolated from the left ventricles, and the myocardial expression of miR-212 and its selected mRNA targets were measured by qRT-PCR. Moreover, left ventricular expression of total-FOXO3, phospho-FOXO3, total-AKT, phospho-AKT, total-mTOR, phospho-mTOR, total-ERK1, total-ERK2, phospho-ERK1, phospho-ERK2, total-AMPK, and phospho-AMPK were measured by Western blot technique.

### 5/6 nephrectomy

Sham operation and 5/6 nephrectomy were performed in two phases as described previously^63^. Anesthesia was induced by intraperitoneal injection of pentobarbital sodium (Euthasol; 40 mg/kg; Produlab Pharma b.v., Raamsdonksveer, The Netherlands). At the first operation, two pieces of sutures (5–0 Mersilk; Ethicon, Sommerville, NJ) were placed around both poles of the left kidney approximately at the 1/3 position. Then the sutures were gently ligated around the kidney. The 1/3 kidney on both ends was excised right beyond the ligatures. Accidental bleeding was alleviated by thermal cauterization. One week after the first operation, animals were anesthetized, and the right kidney was freed from the surrounding adipose tissue as well as the renal capsule, and then it was pulled out of the incision gently. The adrenal gland was gently freed and was placed back into the abdominal cavity. The renal blood vessels and the ureter were ligated, and the right kidney was removed. During sham operations, renal capsules were removed. After the surgeries, the incision was closed with running sutures, and povidone iodide was applied on the surface of the skin. As a post-operative medication, 0.3 mg/kg nalbuphine hydrochloride (Nalbuphine 10 mg/ml; TEVA, Debrecen, Hungary) was administered subcutaneously. Antibiotics (Enroxil, 75 mg; Krka, Slovenia) and analgesics (10 mg/L of nalbuphine hydrochloride, Nalbuphine; TEVA) were administered in tap water for 2 days after both surgeries.

### Transthoracic echocardiography

Cardiac morphology and function were assessed by transthoracic echocardiography at week −1, 4, and 8 (Fig. 1). Rats were anesthetized with 2% isoflurane (Forane, AESICA, Queenborough Limited Kent, UK). Then, the chest was shaved, and the rat was placed in a supine position onto a heating pad. Two-dimensional, M-mode, Doppler, and tissue Doppler echocardiographic examinations were performed by the criteria of the American Society of Echocardiography with a Vivid 7 Dimension ultrasound system (General Electric Medical Systems) using a phased array 5.5–12 MHz transducer (10S probe) as described previously^63–65^. Data of three consecutive heart cycles were analysed (EchoPac Dimension software; General Electric Medical Systems) by an experienced investigator in a blinded manner. The mean values of three measurements were calculated and used for statistical evaluation.

### Blood pressure measurement

To measure arterial blood pressure, a 1.6 F, polyamide, pressure catheter (Scisense Systems Inc, London, ON, Canada) was inserted into the right carotid artery at week 8 under anesthesia (Euthasol; 40 mg/kg; Produlab Pharma b.v., Raamsdonksveer, The Netherlands) in a separated subgroup of animals as we described previously^66^. Blood pressure measurements were performed between 09:00 and 14:00 hours.

### Urine creatinine and total protein levels

At week −1, 4 and 8, a subgroup of animals was placed in metabolic cages (Tecniplast, Italy) for 24 h to collect urine for the measurement of urine creatinine and protein levels to verify the development of CKD. Urine creatinine and urine protein levels were measured by standard laboratory methods as described previously^63^.

### Serum carbamide and creatinine levels

Blood was collected in a subgroup of animals from the saphenous vein at week −1 and 4 and from the thoracic aorta at week 9 to measure serum carbamide (urea) and creatinine levels to verify the development of CKD. Urea and creatinine levels in serum were quantified by kinetic UV method using urease and glutamate dehydrogenase enzymes and Jaffe method, respectively. The reagents and the platform analysers were from Roche Diagnostics (Mannheim, Germany)^63^.

### Creatinine clearance

Creatinine clearance, an indicator of renal function, was calculated according to the standard formula (urine creatinine concentration [μM] × urine volume for 24 h [mL])/(serum creatinine concentration [μM] × 24 × 60 min)^63^. Urine volume and urine creatinine concentration were measured at week 8 and serum creatinine concentration was determined at week 9.

### Serum ion levels

Serum sodium, potassium, calcium, magnesium, phosphate and chloride levels were determined by indirect potentiometry using ion-selective electrodes at week 9. All reagents and instruments were from Roche Diagnostics (Mannheim, Germany)^63^.

### Blood glucose level and OGTT

As described previously, a subgroup of rats was fasted overnight (12h) before blood glucose level measurements to check the development of hyperglycaemia and glucose-intolerance in CKD at week 8^67–70^. Blood samples were collected from the saphenous vein. Blood glucose levels were measured using AccuCheck blood glucose monitoring systems (Roche Diagnostics Corporation, USA, Indianapolis)^67–70^. In case of OGTT, after the measurement of baseline glucose concentrations, 1.5 g/kg body weight glucose was administered *per os* via gavage, and blood glucose levels were checked 30, 60 and 120 min later^67–70^.

### Plasma insulin levels

Plasma insulin levels were measured by an enzyme-linked immunosorbent assay (Mercodia, Ultrasensitive Rat Insulin ELISA) as described previously^67–70^. Blood samples were collected from the saphenous vein at week 8. Insulin ELISA was carried out according to the instructions of the manufacturer from the plasma.

### *Ex vivo* cardiac perfusions and tissue harvesting

Hearts isolated from the animals were perfused for 5 minutes with oxygenated Krebs-Henseleit solution according to Langendorff as described previously^67,69–71^. Coronary flow was measured at the 5^th^ minute. Then the hearts were weighed, left and right ventricles were separated, and a cross-section of the left ventricle at the ring of the papillae was cut and fixed in 10% buffered formalin for histological analysis. Other parts of the left ventricles were freshly frozen and stored at −80 °C until further biochemical measurements. Body and kidney weights were also measured.

### Hematoxylin-eosin staining

Five-μm paraffin-embedded transverse cut sections of the formalin-fixed subvalvular area of the ventricles were stained with hematoxylin-eosin. On these slides, myocardial fibre diameters were measured to verify the development of LVH as described by others^72^. Transverse transnuclear widths (cardiomyocyte diameter) were measured in 100 longitudinally oriented, mono-nucleated cardiomyocytes on left ventricular sections cut on the same plane^72^.

### Picrosirius red staining and image analysis

Five-μm paraffin-embedded transverse cut sections of the formalin-fixed subvalvular area of the ventricles were stained with picrosirius red to assess cardiac fibrosis as described previously^65^. Histological slides were scanned with a *Pannoramic P250* scanner (3D-Histech, Budapest, Hungary) and digital images at the magnification of ×20, ×40 and ×100 were captured. Medium-size vessels and their perivascular connective tissue sheet, the subepicardial and subendocardial areas were avoided as best as possible. The picrosirius red dyed images were analyzed with an in-house developed program as described previously^65^. This program determines the proportion of red pixels of heart sections using two simple color filters. For each Red-Green-Blue (RGB) pixel, the program calculates the color of the pixel in Hue-Saturation-Luminance (HSL) colour space. The first filter is used for detecting red portions of the image. The second filter excludes any white (empty) or light grey (residual dirt on the slide) pixel from further processing using a simple RGB threshold. In this way, the program groups each pixel into one of two sets: pixels considered red and pixels considered green but neither red, nor white, nor grey. Red pixels in the first set correspond to connective tissue and fibrosis. Green pixels in the second set correspond to cardiac muscle. Dividing the number of elements in the first set by the number of elements in both sets gives the proportion of the connective tissue compartment of the heart area examined.

### MicroRNA expression profiling by qRT-PCR

Quantitative RT-PCR was performed with miR-specific primers to monitor miR expression as described earlier^23^. In the case of miRs, RNA was isolated from left ventricles using Trizol reagent (Invitrogen, #15596-018). For quantitative detection of miR-212, TaqMan MicroRNA Reverse Transcription Kit (Applied Biosystems, #4366597), TaqMan miRNA-212 and snoRNA (U64702) Assays (Applied Biosystems, #A25576 and #4427975) and Absolute Blue qPCR Mix (Abgene, #AB-4136/B) were used according to the manufacturer’s instructions. SnoRNA U64702 was used as a control for normalization.

### mRNA expression profiling by qRT-PCR

Quantitative RT-PCR was performed with gene-specific primers to monitor mRNA expression as described previously^67^ (Supplementary Table 1). Target mRNAs of miR-212 associated with cardiac hypertrophy and heart failure were selected in the TargetScan database (Table 6). RNA was isolated using Qiagen RNeasy Fibrous Tissue Mini Kit (Qiagen, #74704) from heart tissue. Briefly, 3 µg of total RNA was reverse transcribed using High-Capacity cDNA Reverse Transcription Kit (Applied Biosystems, #4368814), specific primers and FastStart Essential DNA Green Master (Roche, #06402712001) were used according to the manufacturer’s instructions. Glyceraldehyde-3-phosphate dehydrogenase (*Gapdh*), hypoxanthine phosphoribosyl transferase 1 (*Hprt1*), peptidyl prolyl isomerase A (*Ppia*), and ribosomal protein lateral stalk subunit P2 (*Rplp2*) were used as controls for normalization.

### Matrix metalloprotease 2 (MMP-2) zymography

Cardiac MMP-2 activity was measured from homogenized left ventricular samples to estimate the collagen breakdown in the cardiac extracellular matrix as described previously^73,74^. Briefly, polyacrylamide gels were copolymerized with gelatine, and 40 μg protein was separated by electrophoresis (150 V, 1.5 h) in each lane. Following electrophoresis, gels were washed with 2.5% Triton X-100 and incubated for 20 hours at 37 °C in incubation buffer. Gels were then stained with 0.05% Coomassie Brilliant Blue in a mixture of methanol/acetic acid/water and destained in aqueous 4% methanol/8% acetic acid. Zymograms were digitally scanned, and band intensities were quantified using Quantity One software (Bio-Rad, Hercules, CA).

### Western blot

To investigate gene expression changes at the protein level, standard Western blot technique was used in case of phospho-AKT, AKT, phospho-ERK1/2, ERK1/2, phospho-FOXO3, FOXO3, phospho-AMPK, AMPK, mTOR, and phospho-mTOR with GAPDH loading background^23,75,76^ (Supplementary Figs 1–14). Heart tissue samples (n = 7–8) were homogenized with an ultrasonicator (UP100H Hielscher, Teltow, Germany) in Radio Immunoprecipitation Assay (RIPA) buffer (50 mM Tris–HCl (pH 8.0), 150 mM NaCl, 0.5% sodium deoxycholate, 5 mM ethylenediamine tetra-acetic acid (EDTA), 0.1% sodium dodecyl sulfate, 1% NP-40 (Cell Signaling, Carlsbad, CA, USA) supplemented with protease inhibitor cocktail and phosphatase inhibitors phenylmethane sulfonyl fluoride (PMSF) and sodium fluoride (NaF, Sigma, Saint Louis, MO, USA). The crude homogenates were centrifuged at 15000 × g for 30 min at 4 °C. After quantification of protein concentrations of the supernatants using BCA Protein Assay Kit (Pierce, Rockford, IL, USA), 25 μg of reduced and denaturated protein was loaded. Then sodium dodecyl sulfate–polyacrylamide gel electrophoresis (SDS-PAGE) was performed (10% gel, 90 V, 2 h in case of AKT, ERK1/2, FOXO3, and AMPK, and 6% gel, 50 V, 5 h in case of mTOR) which was followed by transfer of proteins onto a nitrocellulose membrane (20% methanol, 35 V, 2 h in case of AKT, ERK1/2, FOXO3, and AMPK and 10% methanol, 20 V, 16 h, 4 °C in case of mTOR). The efficacy of transfer was checked using Ponceau staining. The membranes were cut horizontally into parts corresponding to the molecular weights of AKT, ERK1/2, FOXO3, AMPK, mTOR and GAPDH and were blocked for 1 h in 5% (w/v) bovine serum albumin (BSA) or milk at room temperature and then incubated with primary antibodies in the concentrations of 1:1000 against phospho-AKT (Ser473, #4060), AKT (#9272), phospho-ERK1/2 (Thr202/Tyr204, #9101 S), ERK1/2 (#9102), phospho-FOXO3 (Ser253; #13129), phospho-AMPK (Thr172; #2535), AMPK (#5831), mTOR (#2972), phospho-mTOR (Ser2448, #2971, overnight, 4 °C, 5% BSA), 1:500 against FOXO3 (#2497, overnight, 4 °C, 5% BSA) and 1:5000 against GAPDH (#2118 overnight, 4 °C, 1% BSA).

Then the membranes were incubated with horseradish peroxidase (HRP)-conjugated goat anti-rabbit secondary antibody 1:2000 (1:1000 for FOXO3, 1:5000 for GAPDH) (Dako Corporation, Santa Barbara, CA, USA; 45 min, room temperature, 1% BSA). After assessment of phosphorylated proteins, the membranes were stripped and reassessed for the total amount of proteins. An enhanced chemiluminescence kit (Cell Signaling, Carlsbad, CA, USA) was used to develop the membranes. The chemiluminescence signals were analyzed and evaluated by Quantity One Software.

### Statistical analysis

The sample size was calculated using the Power and Sample Size Calculation 3.0 free software. Statistical analysis was performed using Sigmaplot 12.0 for Windows (Systat Software Inc). All values are presented as mean ± SEM. Data showed normal distribution unless otherwise indicated. Baseline and different follow-up data including serum metabolite and ion concentrations, and echocardiographic data were compared using Repeated Measures Two-Way ANOVA among the sham and CKD groups. Holm-Sidak test was used as *post hoc* test. Two sample t-test (in case of normal distribution of the data) or Mann Whitney U test (in case of non-normal distribution of the data) were used to determine the effect of CKD on all measured parameters within each time point. P < 0.05 was accepted as a statistically significant difference. In the case of target genes, the analysis of relative gene expression data was performed using the 2^−ΔΔCt^ method. Gene expression ratios with a p-value of <0.05 and fold change of <−1.75 or fold change of >1.75 were considered as repression or overexpression respectively in gene activity.

## Supplementary information

Supplemenentary Figures 1–14 and Table 1

## Data Availability

The datasets generated and analysed during the current study are available from the corresponding author on reasonable request.
