# Supplementary material for: Chronic kidney disease induces left ventricular overexpression of the pro-hypertrophic microRNA-212
Source: Sci Rep. 2019 Feb 4;9:1302. doi: 10.1038/s41598-018-37690-5 (PMC6362219; doi:10.1038/s41598-018-37690-5)
Supplement: Supplementary file 1 — Supplemenentary Figures 1–14 and Table 1 [file 41598_2018_37690_MOESM1_ESM.pdf]

Chronic kidney disease induces left ventricular overexpression of the pro-hypertrophic microRNA-212

Márta Sárközy, Renáta Gáspár, Ágnes Zvara, Andrea Siska, Bence Kővári, Gergő Szűcs, Fanni Márványkövi, Mónika G. Kovács, Petra Diószegi, László Bodai, Nóra Zsindely, Márton Pipicz, Kamilla Gömöri, Krisztina Kiss, Péter Bencsik, Gábor Cserni, László G. Puskás, Imre Földesi, Thomas Thum, Sándor Bátkai, Tamás Csont

Supplementary Figures 1-14

Supplementary Figure 1

Uncropped, full-length Western blot image (Total-FOXO3/GAPDH)

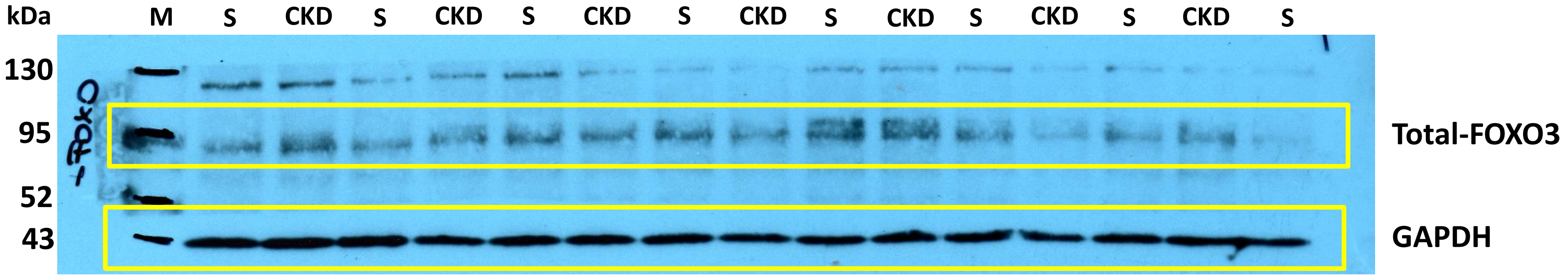

CKD: chronic kidney disease; M: marker; S: sham.
(The marker was a high-range prestained marker)

Supplementary Figure 2

Uncropped, full-length Western blot images
Phospho-FOXO3/GAPDH
(different exposition times)

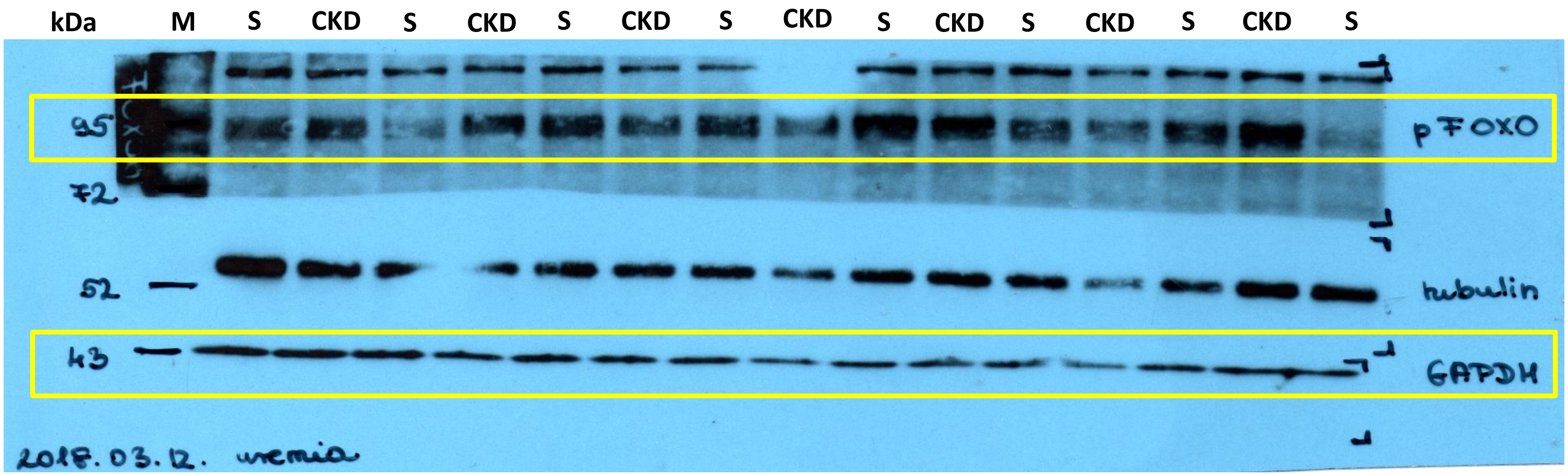

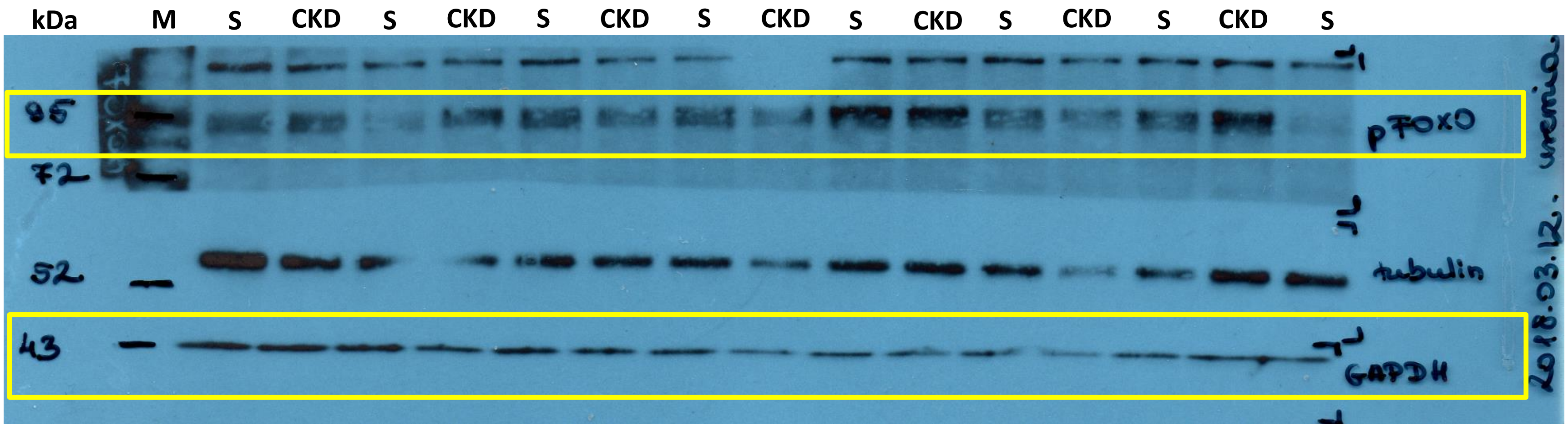

CKD: chronic kidney disease; M: marker; pFOXO: phospho-FOXO3, S: sham.
(The marker was a high-range prestained marker)

Supplementary Figure 3

Uncropped, full-length Western blot images
Total-Akt/GAPDH

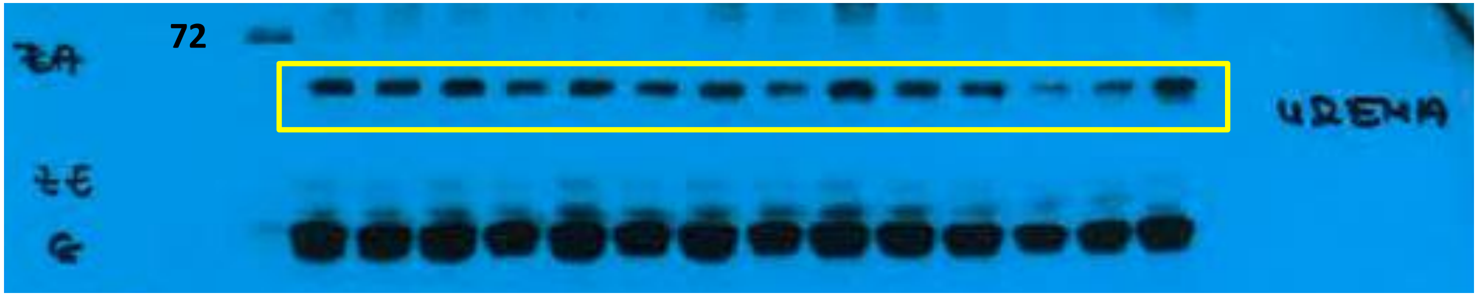

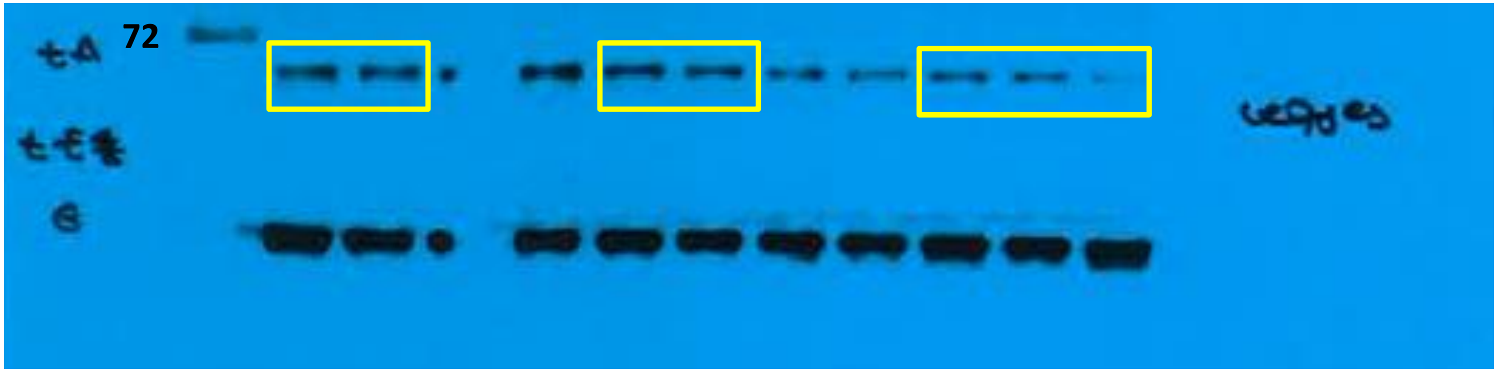

CKD: chronic kidney disease; M: marker; PC: technical positive control; S: sham.
(The marker was page ruler prestained ladder)

Supplementary Figure 4

Uncropped, full-length Western blot images
Total-Akt/GAPDH
(at a different exposition time than on Figure 3)

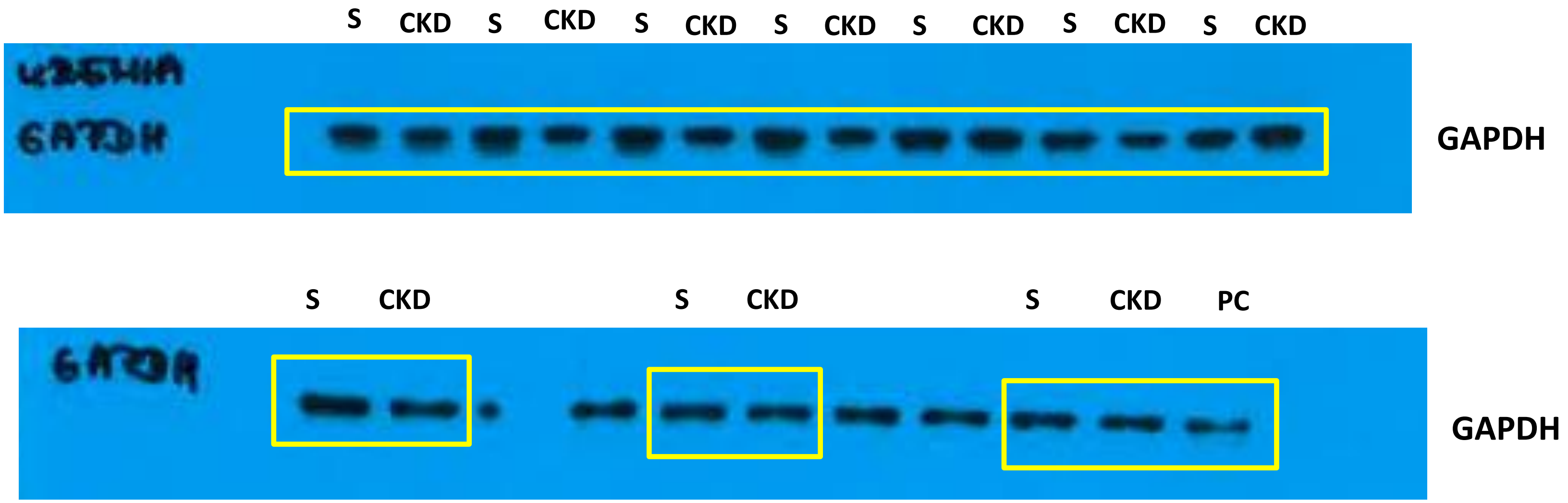

CKD: chronic kidney disease; M: marker; PC: technical positive control; S: sham.
(The marker was page ruler prestained ladder)

Supplementary Figure 5

Uncropped, full-length Western blot images
Phospho-Akt/GAPDH

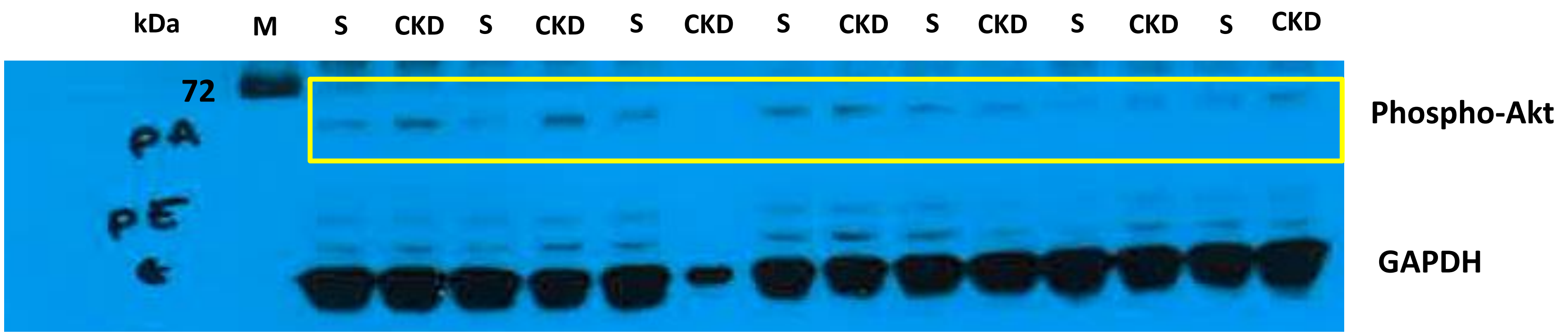

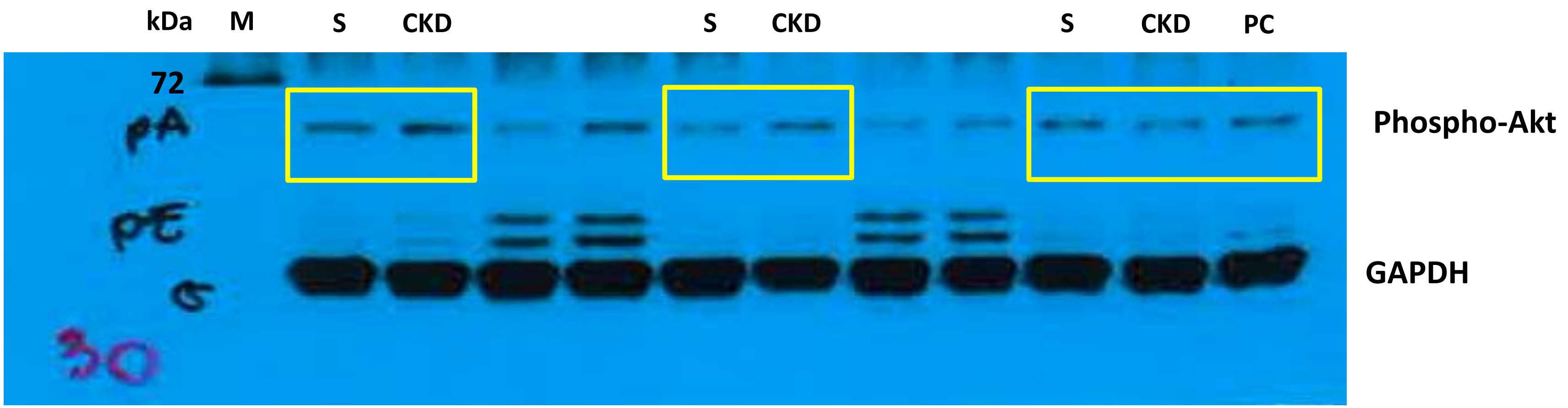

CKD: chronic kidney disease; M: marker; PC: technical positive control; S: sham.
(The marker was page ruler prestained ladder)

Supplementary Figure 6

Uncropped, full-length Western blot images
Phospho-Akt/GAPDH
(at a different exposition time than on Supplementary Figure 5)

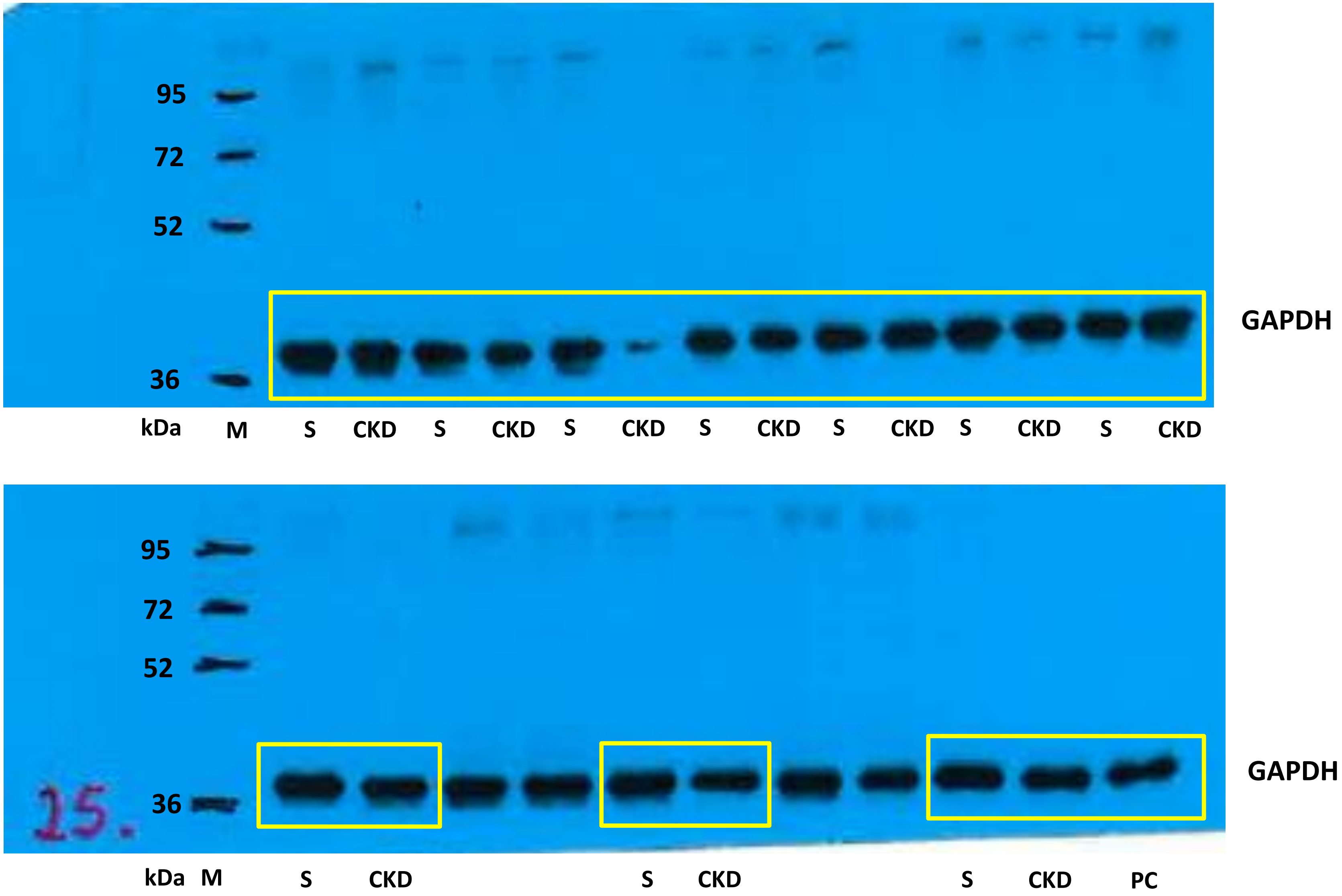

CKD: chronic kidney disease; M: marker; PC: technical positive control; S: sham.
(The marker was page ruler prestained ladder)

Supplementary Figure 7

Uncropped, full-length Western blot images
Phospho-ERK 1 2/GAPDH

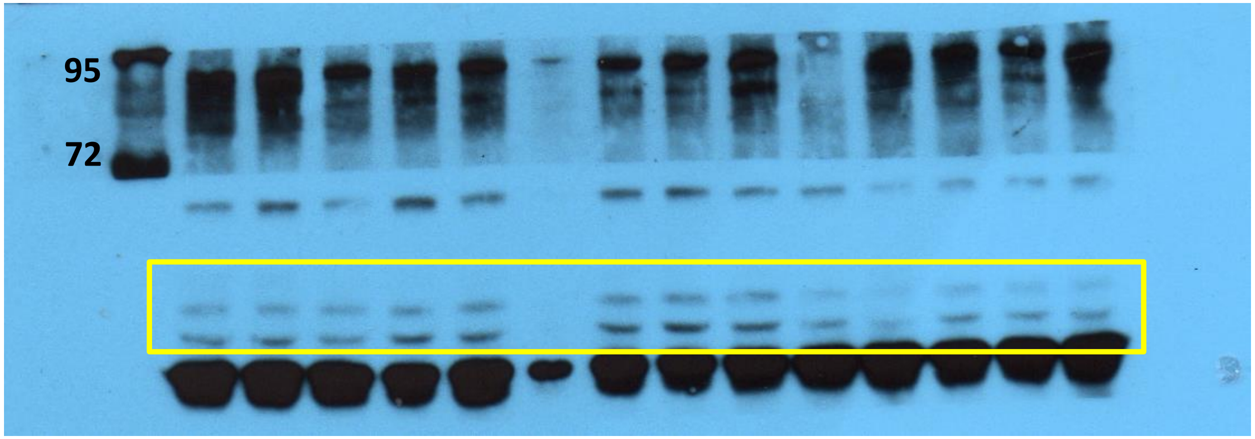

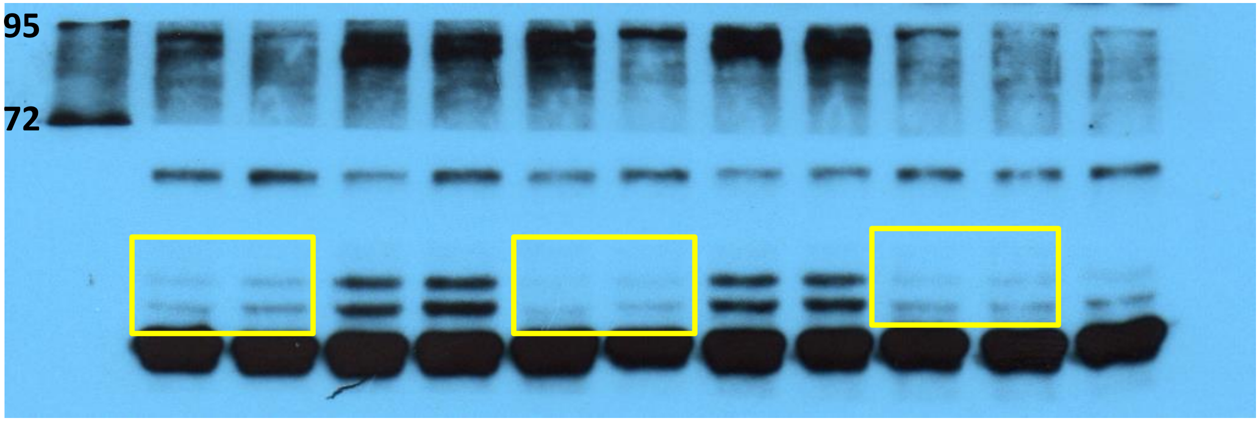

CKD: chronic kidney disease; M: marker; PC: technical positive control; S: sham.
(The marker was page ruler prestained ladder)

Supplementary Figure 8

Uncropped, full-length Western blot images
Phospho-ERK 1 2/GAPDH
(at a different exposition time than on Figure 7)

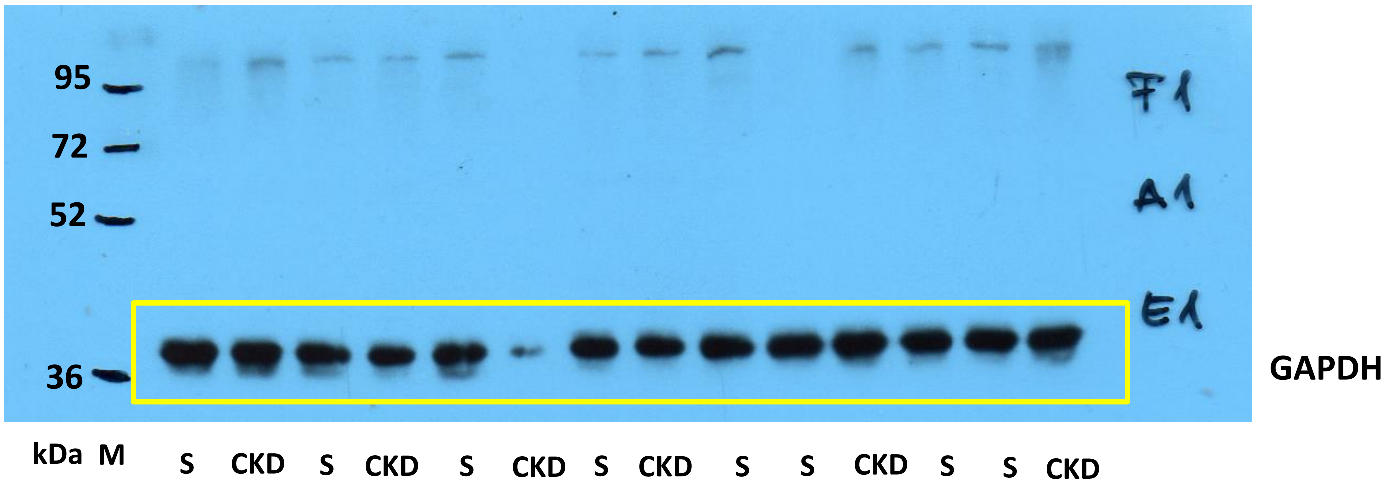

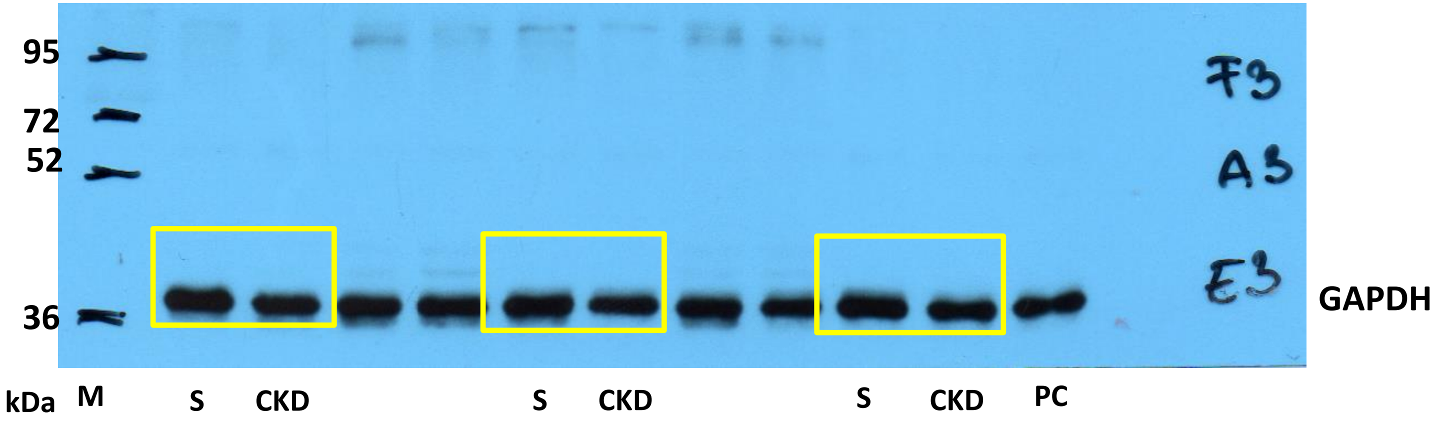

CKD: chronic kidney disease; M: marker; PC: technical positive control; S: sham.
(The marker was page ruler prestained ladder)

Supplementary Figure 9

Uncropped, full-length Western blot images
Total-ERK 1 2/GAPDH

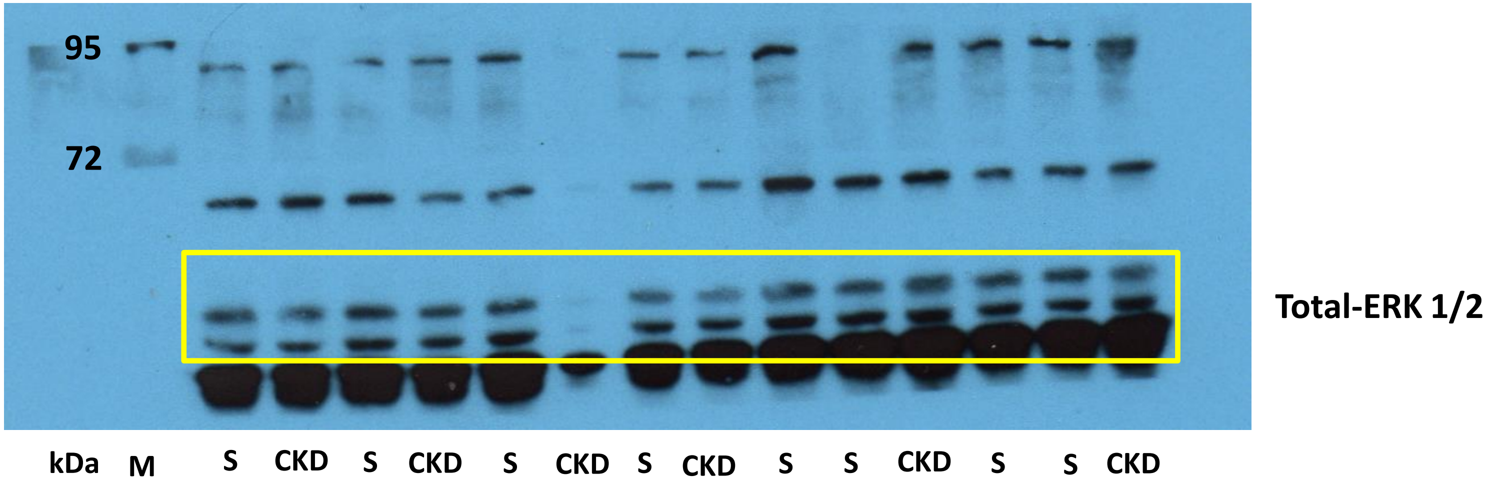

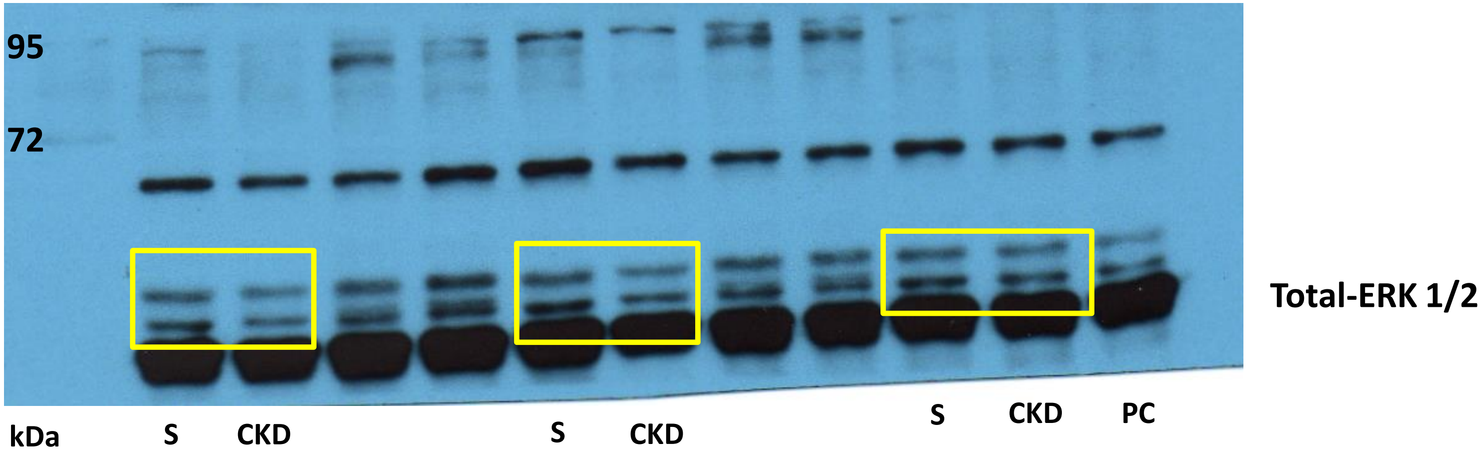

CKD: chronic kidney disease; M: marker; PC: technical positive control; S: sham.
(The marker was page ruler prestained ladder)

Supplementary Figure 10

Uncropped, full-length Western blot images
Total-ERK 1 2/GAPDH
(at a different exposition time than on Figure 9)

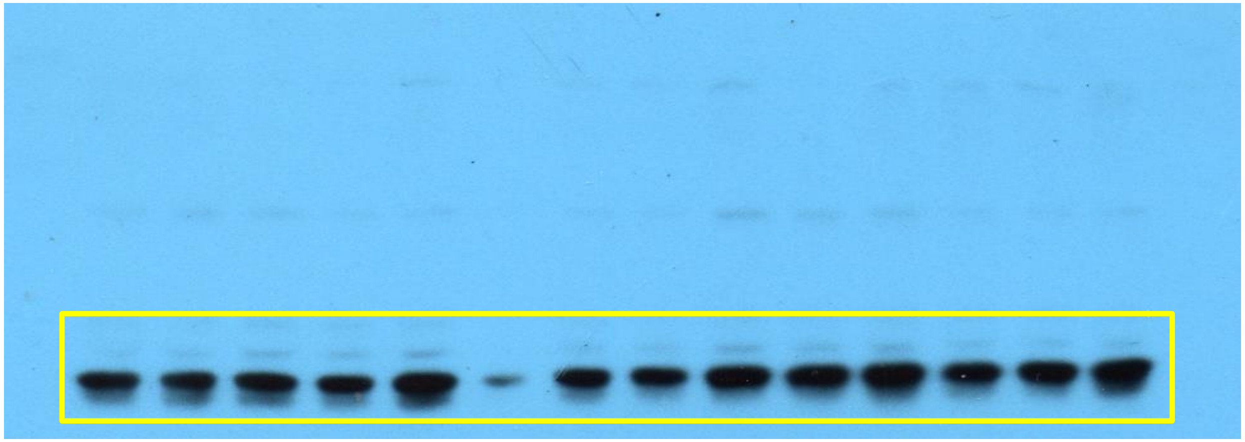

GAPDH

S CKD S CKD S CKD S CKD S S CKD S S CKD

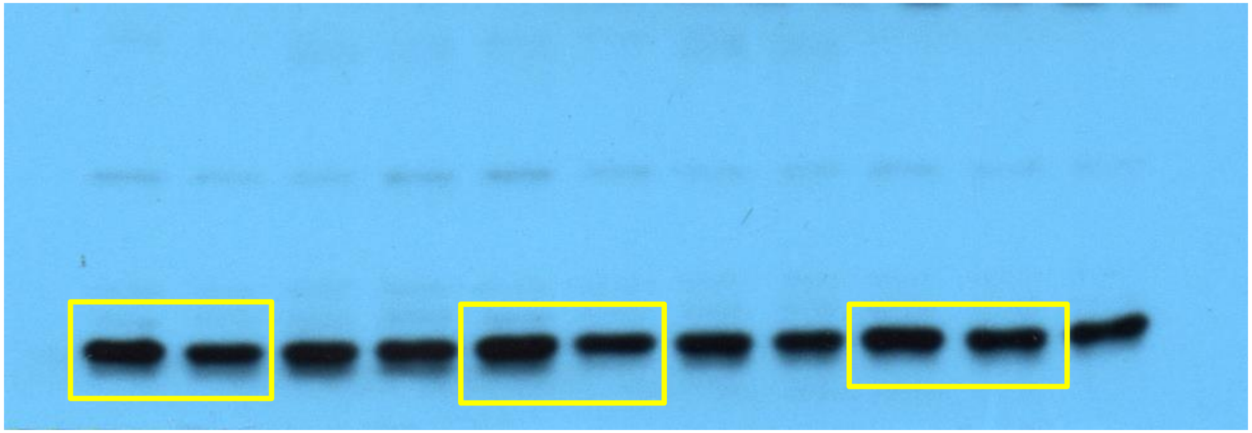

GAPDH

S CKD S CKD S CKD PC

CKD: chronic kidney disease; M: marker; PC: technical positive control; S: sham.
(The marker was page ruler prestained ladder)

Supplementary Figure 11

Uncropped, full-length Western blot images
Total-AMPK/GAPDH

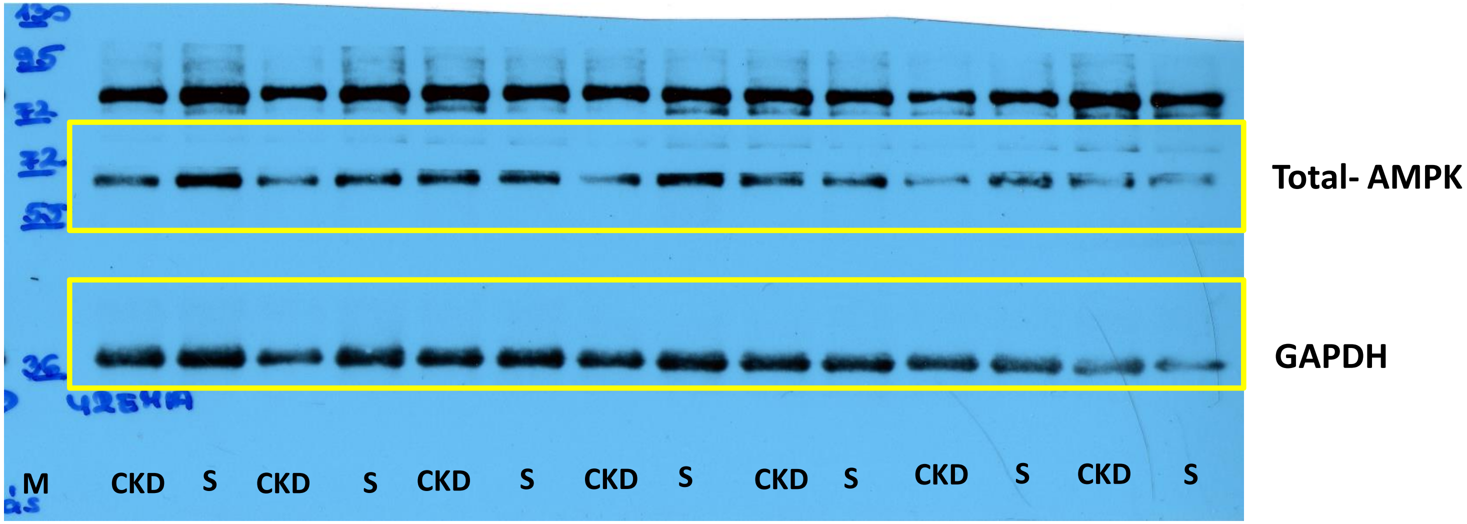

CKD: chronic kidney disease; M: marker; S: sham.
(The marker was page ruler prestained ladder)

Supplementary Figure 12

Uncropped, full-length Western blot images
Phospho - AMPK/GAPDH

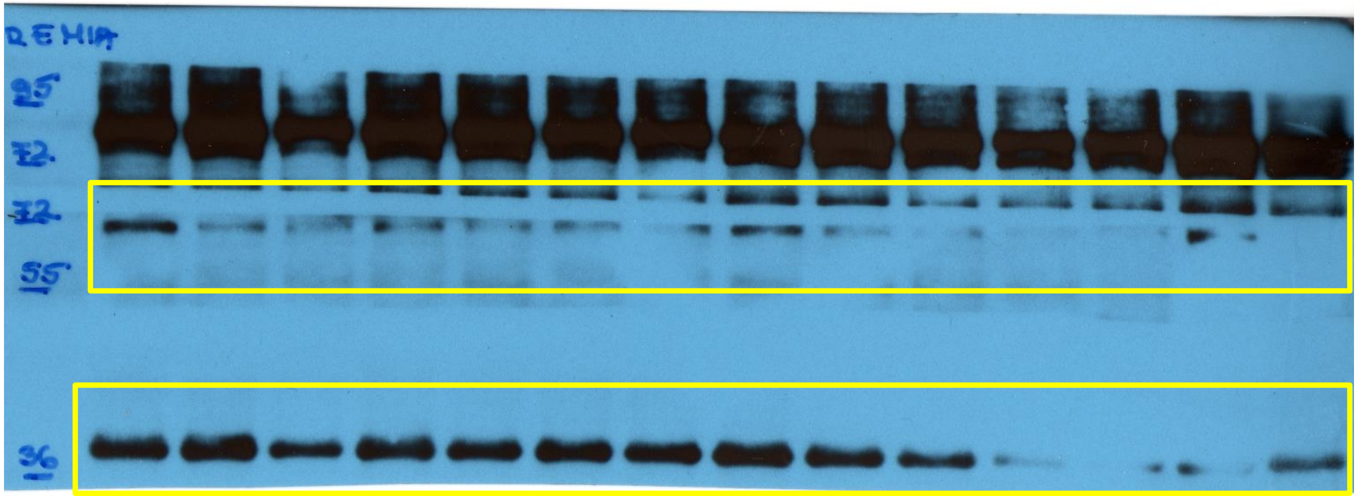

Phospho- AMPK

GAPDH

M CKD S CKD S CKD S CKD S CKD S CKD S CKD S

CKD: chronic kidney disease; M: marker; S: sham.
(The marker was page ruler prestained ladder)

Supplementary Figure 13

Uncropped, full-length Western blot images
Total- mTOR/GAPDH

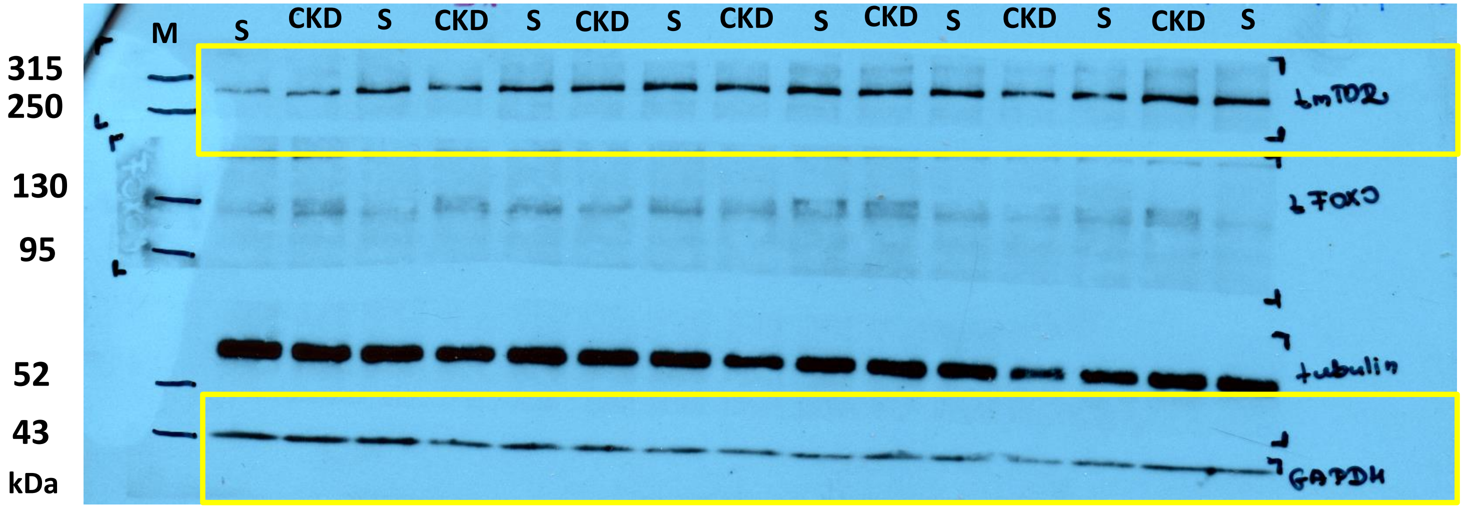

CKD: chronic kidney disease; M: marker; S: sham.
(The marker was a high-range prestained marker)

Supplementary Figure 14

Uncropped, full-length Western blot images
Phospho- mTOR/GAPDH

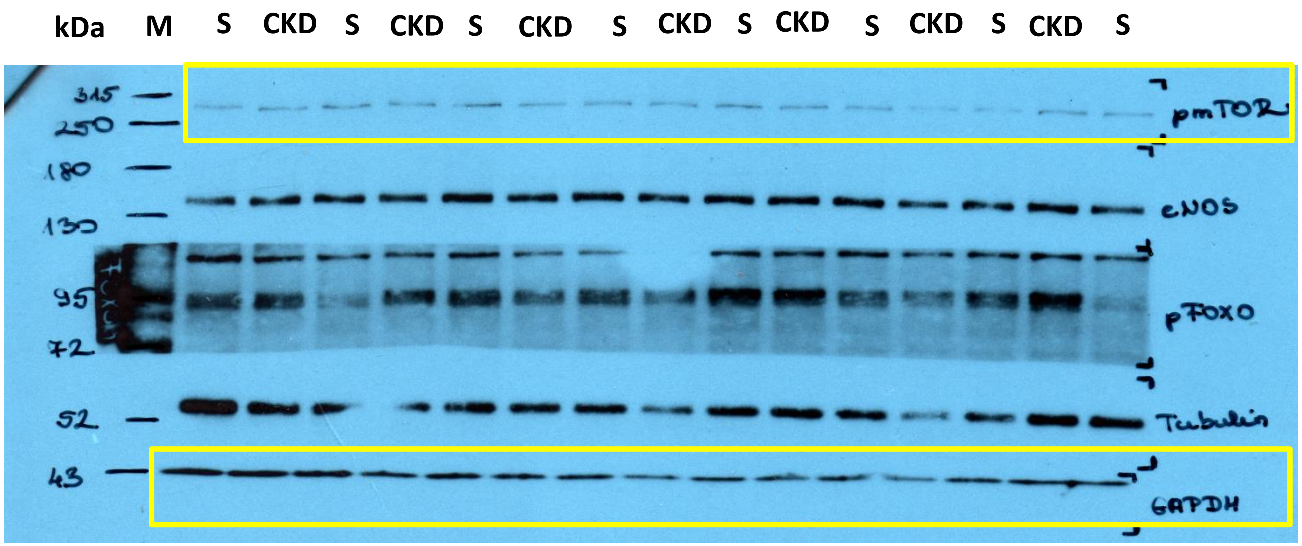

CKD: chronic kidney disease; M: marker; S: sham.
(The marker was a high-range prestained marker)

Supplementary Table

Chronic kidney disease induces left ventricular overexpression of the pro-hypertrophic microRNA-212

Márta Sárközy[1#], Renáta Gáspár[1], Ágnes Zvara[2], Andrea Siska[3], Bence Kővári[4], Gergő Szűcs[1], Fanni Márványkövi[1], Mónika G. Kovács[1], Petra Diószegi[1], László Bodai[5], Nóra Zsindely[5], Márton Pipicz[1], Kamilla Gömöri[6], Krisztina Kiss[6], Péter Bencsik[6], Gábor Cserni[4], László G. Puskás[2], Imre Földesi[3], Thomas Thum[7], Sándor Bátkai[7], Tamás Csont[1]

[1]Metabolic Diseases and Cell Signaling Group, Department of Biochemistry, Faculty of Medicine, University of Szeged, Dóm tér 9, Szeged, H-6720, Hungary
[2]Laboratory for Functional Genomics, Institute of Genetics, Biological Research Center of the Hungarian Academy of Sciences, Temesvári krt. 62, H-6701, Szeged, Hungary
[3]Department of Laboratory Medicine, Faculty of Medicine, University of Szeged, Semmelweis utca 6, Szeged, H-6725, Hungary,
[4]Department of Pathology, University of Szeged, Állomás utca 1, Szeged, H-6725, Hungary
[5]Department of Biochemistry and Molecular Biology, Faculty of Science and Informatics, University of Szeged, Közép fasor 52, Szeged, H-6726, Hungary
[6]Cardiovascular Research Group, Department of Biochemistry, Faculty of Medicine, University of Szeged, Dóm tér 9, Szeged, H-6720, Hungary
[7]IMTTS, Hannover Medical School, Carl-Neuberg Strasse 1, Hannover, 30625, Germany

[#]Corresponding author

Supplementary table 1 Primer sequences.

Gene name	Gene symbol	Forward primer sequence	Reverse primer sequence
Peptidylprolyl isomerase A (cyclophilin A)	*Ppia*	tgctggaccaaacacaaatg	caccttcccaaagaccacat
Hypoxanthine phosphoribosyltransferase 1	*Hprt1*	gaccggttctgtcatgtcg	acctggttcatcatcactaatcac
Ribosomal protein, large P2	*Rplp2*	agcgccaaagacatcaagaa	tcagctcactgatgaccttgtt
Glyceraldehyde-3-phosphate dehydrogenase (GAPDH)	*Gapdh*	gaagggctcatgaccacagt	ggatgcagggatgatgttct
Natriuretic peptide A (ANP)	*Nppa*	gccggtagaagatgaggtca	gggctccaatcctgtcaatc
Natriuretic peptide B (BNP)	*Nppb*	tctgctcctgcttttcctta	gaactatgtgccatcttgga
Myosin, Heavy Polypeptide 6, Cardiac Muscle, Alpha (α-MHC)	*Myh6*	ggaagagcgagcggcgcatcaagg	ctgctggacaggttattcctca
Myosin, Heavy Polypeptide 7, Cardiac Muscle, Beta (β-MHC)	*Myh7*	gccaacaccaacctgtccaagttc	ttcaaaggctccaggtctcagggc
Myocyte enhancer factor 2C (predicted)	*Mef2c*	gcagccatagtccggtga	ccccttcctctcttagcc
Myocyte enhancer factor 2D	*Mef2d*	cccactgcctacaacacaga	aggctggtaaggaggaaagc
Protein Kinase B Alpha	*Akt1*	tcacctctgagaccgacacc	actggctgagtaggagaactgg
Protein phosphatase 3 catalytic subunit alpha	*Ppp3ca*	tgaggctgaaaaagcaatacg	aaaccctttgcctcttcaaaa
Protein phosphatase 3 catalytic subunit beta	*Ppp3ca*	tcagaagaagatggatttgacg	tgctcggatcttgttcctg
Nuclear Factor Of Activated T-Cells, Cytoplasmic, Calcineurin-Dependent 4	*Nfatc4*	gggggctgtcaaggctgctc	gcgcccgatgtctgtctcacc
Muscle Atrophy F-Box Protein (atrogin 1)	*Fbx32*	ccatcaggagaagtggatctatgtt	gttcatgaagttcttttgggcgatgc
Myocyte-Enriched Calcineurin-Interacting Protein 1 (MCIP1.4)	*Rcan1*	agctccctgattgcctgtgt	tttggccctggtctcacttt
Forkhead box O3	*Foxo3*	gatggtgcgctgtgtgccctac	ccaagagctcttgccagtccctt
Mitogen activated protein kinase 1 (ERK2)	*Mapk1*	tctgcaccgtgacctcaa	gcaaggccaaagtcacaga
Myocyte enhancer factor 2a	*Mef2a*	gcacacagagcaccttgtaga	ttaggagacaagtagtccaaggaag
Protein kinase AMP-activated catalytic subunit alpha 2 (AMPK)	*Prkaa2*	gacaatcggagctatcttctagactt	aggtgttgaagaaccagacctc
DnaJ heat shock protein family (Hsp40) member A2	*Dnaja2*	aggtgtgcgcattatgataaga	cggtctttttcattgatgacct
Sirtuin 1, transcript variant X1 (predicted)	*Sirt1*	ttcgtggagatatttttaatcaggt	ctggtaagttttcaccaaagaagac
Phosphatase and tensin homolog	*Pten*	catgagcgagttggtcaaga	ccatgctgtgctggttca
